# Understanding the prebiotic potential of different dietary fibers using an *in vitro* continuous adult fermentation model (PolyFermS)

**DOI:** 10.1038/s41598-018-22438-y

**Published:** 2018-03-12

**Authors:** Sophie A. Poeker, Annelies Geirnaert, Laura Berchtold, Anna Greppi, Lukasz Krych, Robert E. Steinert, Tomas de Wouters, Christophe Lacroix

**Affiliations:** 1 Laboratory of Food Biotechnology, Department of Health Sciences and Technology, ETH Zurich, Schmelzbergstrasse 7, Zürich, Switzerland; 2 Department of Food Science, Faculty of Science, University of Copenhagen, Copenhagen, Denmark; 3 DSM Nutritional Products Ltd., R&D Human Nutrition and Health, Basel, Switzerland

## Abstract

Consumption of fermentable dietary fibers (DFs), which can induce growth and/or activity of specific beneficial populations, is suggested a promising strategy to modulate the gut microbiota and restore health in microbiota-linked diseases. Until today, inulin and fructo-oligosaccharides (FOS) are the best studied DFs, while little is known about the gut microbiota-modulating effects of β-glucan, α-galactooligosaccharide (α-GOS) and xylo-oligosaccharide (XOS). Here, we used three continuous *in vitro* fermentation PolyFermS model to study the modulating effect of these DFs on two distinct human adult proximal colon microbiota, independently from the host. Supplementation of DFs, equivalent to a 9 g daily intake, induced a consistent metabolic response depending on the donor microbiota. Irrespective to the DF supplemented, the *Bacteroidaceae-Ruminococcaceae* dominated microbiota produced more butyrate (up to 96%), while the *Prevotellaceae-Ruminococcaceae* dominated microbiota produced more propionate (up to 40%). Changes in abundance of specific bacterial taxa upon DF supplementation explained the observed changes in short-chain fatty acid profiles. Our data suggest that the metabolic profile of SCFA profile may be the most suitable and robust read-out to characterize microbiota-modulating effects of a DF and highlights importance to understand the inter-individual response to a prebiotic treatment for mechanistic understanding and human application.

## Introduction

The human gut microbiota is composed of around 10^14^ bacterial cells that belong to more than 1000 species^1^, dominated by members belonging to the two phyla, Firmicutes and Bacteroidetes^1,2^. Diet is known to strongly influence the composition of the gut microbiota as well as metabolites, dominated by the canonical short chain fatty acids (SCFAs) acetate, propionate and butyrate^3,4^. Among healthy individuals, compositional and functional properties of the microbiome vary substantially, leading to highly variable responses to dietary interventions^5–9^. Baseline bacterial composition of the host microbiome has repeatedly been observed to be a key factor to explain responses of the gut microbiota to different dietary interventions^10–12^. Within the collective genome of several millions genes, the microbiome harbors the capacity of primary degradation of substrates by specialized bacteria, cross-feeding and competition, making the stratification of microbiota response profiles a major challenge in the field^1,10^.

Around 40 g of complex carbohydrates reach the colon each day after escaping breakdown by host enzymes^9,13,14^. Endogenous enzymes are unable to degrade numerous complex carbohydrates and plant polysaccharides^4^. Non-digestible dietary fibers have been shown to have a beneficial effect on intestinal wellbeing acting as bulking agent and substrates for growth and activity of specific endogenous bacterial populations within the gastrointestinal tract (GIT)^15^. The breakdown of the complex carbohydrates by the gut microbiota is a key factor for the stability and diversity of the intestinal ecosystem yielding energy not only for the host, but also for its microbiota. The presence of end metabolites such as the SCFAs acetate, propionate and butyrate, and absence of intermediate metabolite accumulation, such as for lactate, formate and succinate, are generally recognized as markers for a healthy microbiome^16^. Metabolism of fibers is occurring in the colon, especially in the proximal colon resulting in an increased production of organic acids and a decrease in luminal pH of 5.5–5.9^17^. Acidification but more importantly the production of intermediate and end-metabolites have important consequences for the microbial composition, the establishment of key bacterial interactions and the proper functioning of host physiology^18,19^. Microbial SCFAs impact on gut health, as energy source for the intestinal epithelium and epigenetic factor influencing immune response, epithelial integrity, electrolytes re-absorption and gut motility. Butyrate is an energy substrate used by colonocytes, while acetate and propionate reach systemic circulation and affect metabolism and function of peripheral organs (e.g. liver, pancreas, brain, muscle)^4,20,21^. Therefore fiber modulation of microbiota composition and functions has crystallized as a promising strategy to promote gut and host health.

Dietary fibers (DFs) as main substrate for the gut microbiota are key factors of the microbial network in the gut. In particular inulin and fructo-oligosaccharides (FOS) have repeatedly been shown to selectively modulate the gut microbiota *in vitro* and *in vivo* with benefits for host health (“prebiotic effect”)^22–27^. However, the lack of specific responses of bacterial groups has posed an important challenge in understanding the prebiotic mechanisms of most fibers^12,28^. Multiple studies aiming at identification of novel prebiotics, such as β-glucans, galacto-oligosaccharides (GOS) and xylo-oligosaccharides (XOS), have encountered the same challenge of functional redundancy within phylogenetically diverse bacterial groups and limited understanding of the metabolite functions.

*In vitro* fermentation models are powerful approaches to investigate gut microbiota functionality without host effects in a highly controlled environment^29^. These models allow the strict control of physiologic parameters, such as retention time, pH, temperature and anaerobiosis, and medium composition used to mimic the diet. Colonic models from simple short-term batch fermentations to multistage long-term continuous flow models were developed^30–32^. Continuous models further control the medium flow rate, for culturing of microbiota in steady-state conditions, allowing a fiber to develop its full effect along the entire trophic chain, thereby increasing the physiological relevance of the experiment. In particular, continuous fermentation systems with immobilized gut microbiota were shown to simulate the high-cell density, biodiversity and long-term stability of the intestinal microbiota^32^. This prevents washout of less competitive bacteria and ensures the repeated exposure of a single microbiota to different fibers^29,30,32^. The PolyFermS model allows the parallel testing of different treatments on singular microbiota.

The aim of this study was therefore to investigate the effects of novel potential prebiotics on healthy adult gut microbiota using PolyFermS *in vitro* continuous colonic fermentation model mimicking the adult proximal colon microbiota. Immobilization of two distinct fecal microbiota obtained from two healthy adult donors was performed. The two microbiota were propagated and stabilized in an inoculum reactor (IR) seeded with microbiota immobilized in polymer gel beads allowing continuous and prolonged culture of the microbiome^31,33^. The model design allowed parallel testing of four different dietary fiber supplementations (β-glucan, XOS, α-GOS and inulin) compared to a control reactor with no supplemented fiber, all inoculated with the same microbiota produced in IR. The effect of dietary fibers derived from plants was tested at a physiological concentration mimicking a daily intake of 9 g fibers in test reactors for 5–7 days to reach pseudo-steady state conditions. Microbiota composition and diversity was monitored with 16S rRNA gene amplicon sequencing, and SCFAs analysis by HPLC.

## Results

### Donor selection and transfer to PolyFermS

**Figure 1:**
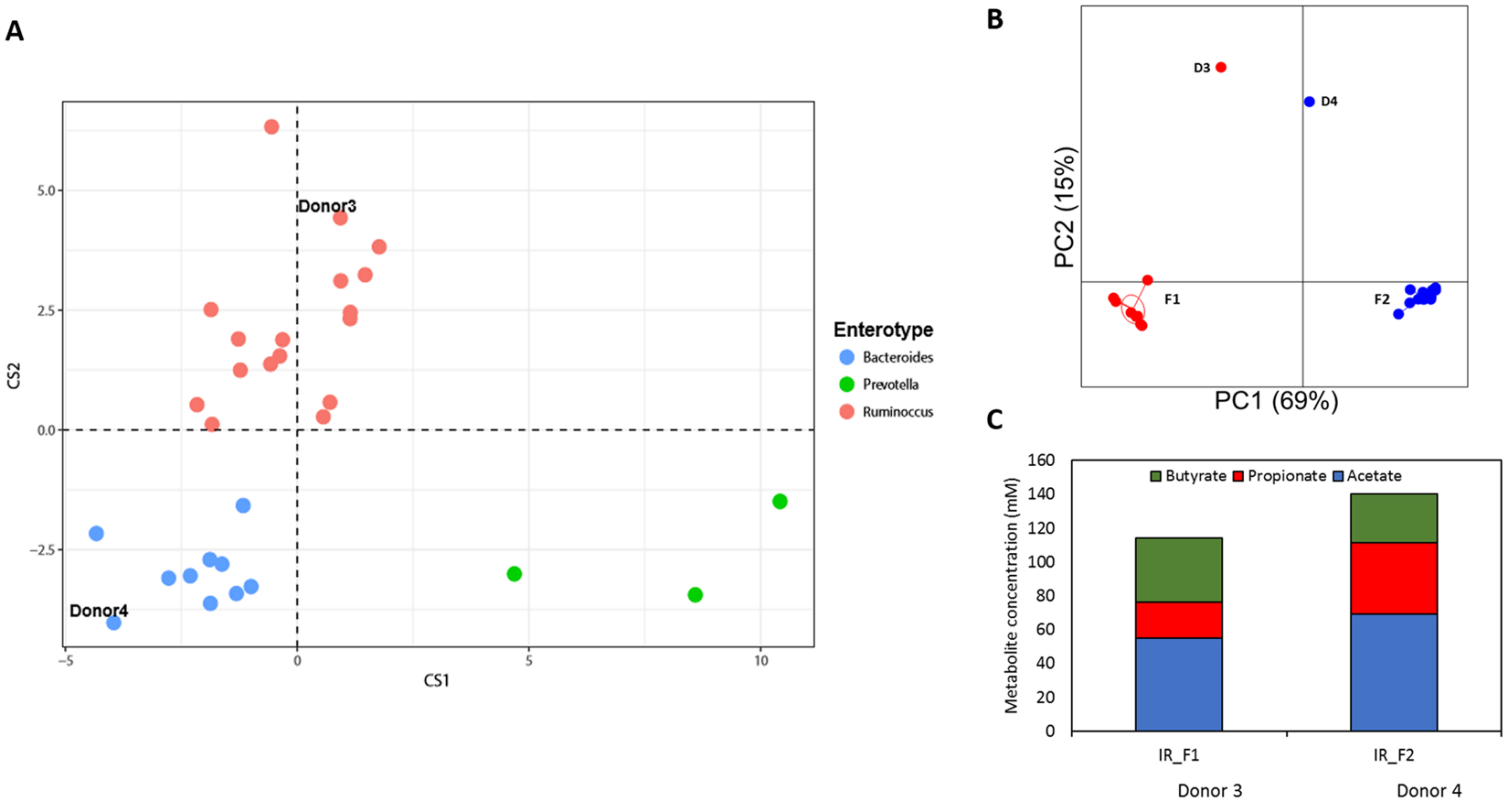
(**A**) Variation in fecal microbiota among the 30 healthy individuals represented in an enterotype plot for 16S rRNA gene amplicon sequence dataset generated as described on http://enterotyping.embl.de/. (**B**) Principle Coordinate Analysis based on the weighted UniFrac distance matrix on OTU level generated from fecal and non-treated PolyFermS microbiota of fermentation 1 (F1 with donor 3 microbiota) and fermentation 2 (F2 with donor 4 microbiota), showing conservation of the two distinct donor microbiota profiles in PolyFermS model. Microbiota included from inoculum reactor (IR) and control reactor (CR) from F1 on day 12, day 19, day 24 and day 31 and for F2 on day 8, day 14, day 19, day 25, day 31 and day 37. (**C**) Bar-plot representation of mean acetate (blue), propionate (red) and butyrate (green) concentrations (mM) of PolyFermS effluent samples of IR during stable operation phase and connection with experimental reactors (IR_F1: day 1–day 36; IR_F2: day 1–day 42).

Fecal microbiota of 30 healthy individuals were screened using 16S rRNA gene amplicon sequencing to select two fecal donors with distinct taxonomic profile for dietary fiber supplementation study. Fecal microbiota variation across the donors was stratified into enterotypes *Ruminococcus*, *Bacteroides* and *Prevotella* as decribed by Arumugam *et al*.^34^ (Fig. 1A). Two donors, D3 and D4, were selected based on the difference in their dominant microbial taxa. The microbiota composition of both donors was representative for a healthy human microbiota (Supplementary Table S2), with *Firmicutes* (D3: 51%; D4: 57%) and *Bacteroidetes* (D3: 26%; D4: 32%) as the dominant bacterial phyla. Donors differed from each other on family level with D3 fecal microbiota characterized by higher levels of *Bacteroidaceae* (D3: 17%; D4: 8%) and presence of *Verrucomicrobiaceae* (D3: 6%; D4: < 0%), and archaeal family *Methanobacteriaceae* (D3: 9%; D4: 2%). Whereas D4 fecal microbiota was characterized by higher levels of *Prevotellaceae* (D4: 17%; D3: 0.1%) and *Lachnospiraceae* (D4: 13%; D3: 7%) compared to D3 fecal microbiota (Supplementary Table S2B). For assessing the α-diversity, the Shannon index for both fecal microbiota was calculated and both donors had a comparable Shannon index (D3: 5.8 ± 0.9; D4: 5.8 ± 0.9; Supplementary Table S3).

Fecal microbiota of both donors were studied in the PolyFermS continuous intestinal fermentation model operated with conditions mimicking the proximal colon microbiota. The model is composed of an inoculum reactor (IR), containing immobilized human adult fecal microbiota used to continuously inoculate several second stage control and experimental reactors mounted in parallel. The second stage reactors with the same complete microbiota allow comparing different treatments with a control. During 49 days of continuous operation of IRs inoculated with different donor microbiota, the microbial stability was assessed on phylogenetic and metabolic levels. The Shannon diversity, taking in account both abundance and evenness of species present in a sample, was reduced in the *in vitro* model compared with fecal microbiota (Supplementary Table S3). The mean Shannon index of IR and second stage untreated microbiota (CR) was 4.3 ± 0.1 for F1, containing D3 microbiota (Shannon index 5.8 ± 0.9), and 3.6 ± 0.2 for F2, containing D4 microbiota (Shannon index 5.8 ± 0.9). On family level the microbiota composition within each fermentation reflected well its specific donor microbiota (Supplementary Table S4 and Supplementary Table S5) as previously observed in similar PolyFermS setup^32^. Within F1 microbiota of IR and untreated control reactor, Firmicutes (56%) and Bacteroidetes (26%) were the predominant phyla. In correspondence with the fecal D3 microbiota, F1 microbiota was characterized by high levels of bacterial families *Bacteroidaceae* (IR: 23 ± 5%; CR: 29 ± 6%) and *Ruminococcaceae* (IR: 18 ± 3%; CR: 11 ± 7%). Whereas F2 microbiota was dominated by Bacteroidetes (58%) over Firmicutes (36%) and characterized by high levels of *Prevotellaceae* (IR: 53 ± 6%; CR: 53 ± 4%) and *Ruminococcaceae* (IR: 19 ± 4%; CR: 18 ± 2%), also in correspondence with its donor fecal microbiota. Some bacterial families were detected at different abundances *in vitro* compared to the fecal sample. For example, in F1 increased abundances of *Acidaminococcaceae* (12 ± 1%; 0.3% in fecal sample) and *Enterobacteriaceae* (15 ± 4%; undetected in fecal sample) and in F2 *Prevotellaceae* (36 ± 6%; 17.2% in fecal sample) and unclassified Lactobacillales (4 ± 1%; undetectable in fecal sample) were detected (Supplementary Table S2 and Supplementary Table S5). A clear spatial separation and clustering of F1 and F2 microbiota was observed by Principle Coordinate Analysis (PCoA) on weighted UniFrac distance matrix on OTU level, indicating a distinct and stable microbial profile of the two donor microbiota *in vitro* (Fig. 1B).

Bacterial fermentation activity was monitored by SCFA analysis of fermentation effluents of PolyFermS reactors along the complete fermentation period. After the initial colonization-stabilization period of 12 and 15 days, total SCFA concentrations in IR effluents were stable with 153 (±9) mM for 20 days in F1 and 123 (±10) mM for 36 days in F2, respectively. Acetate was the predominant SCFA produced in both fermentations (F1: 55 ± 6 mM; F2: 69 ± 4 mM). Butyrate and propionate levels differed between both fermentations with F1 characterized by higher butyrate levels (F1: 38 ± 4 mM and F2: 29 ± 6 mM) and F2 characterized by higher propionate levels (F1: 21 ± 3 mM and F2: 42 ± 5 mM) (Fig. 1C). The metabolic profiles of IR over the whole fermentation periods of 36 and 42 days for F1 and F2, respectively, showed stable concentrations of the main SCFAs (Supplementary Figure S2).

### Dietary fiber supplementation induces different metabolic and microbial responses *in vitro* depending on donor microbiota

**Figure 2:**
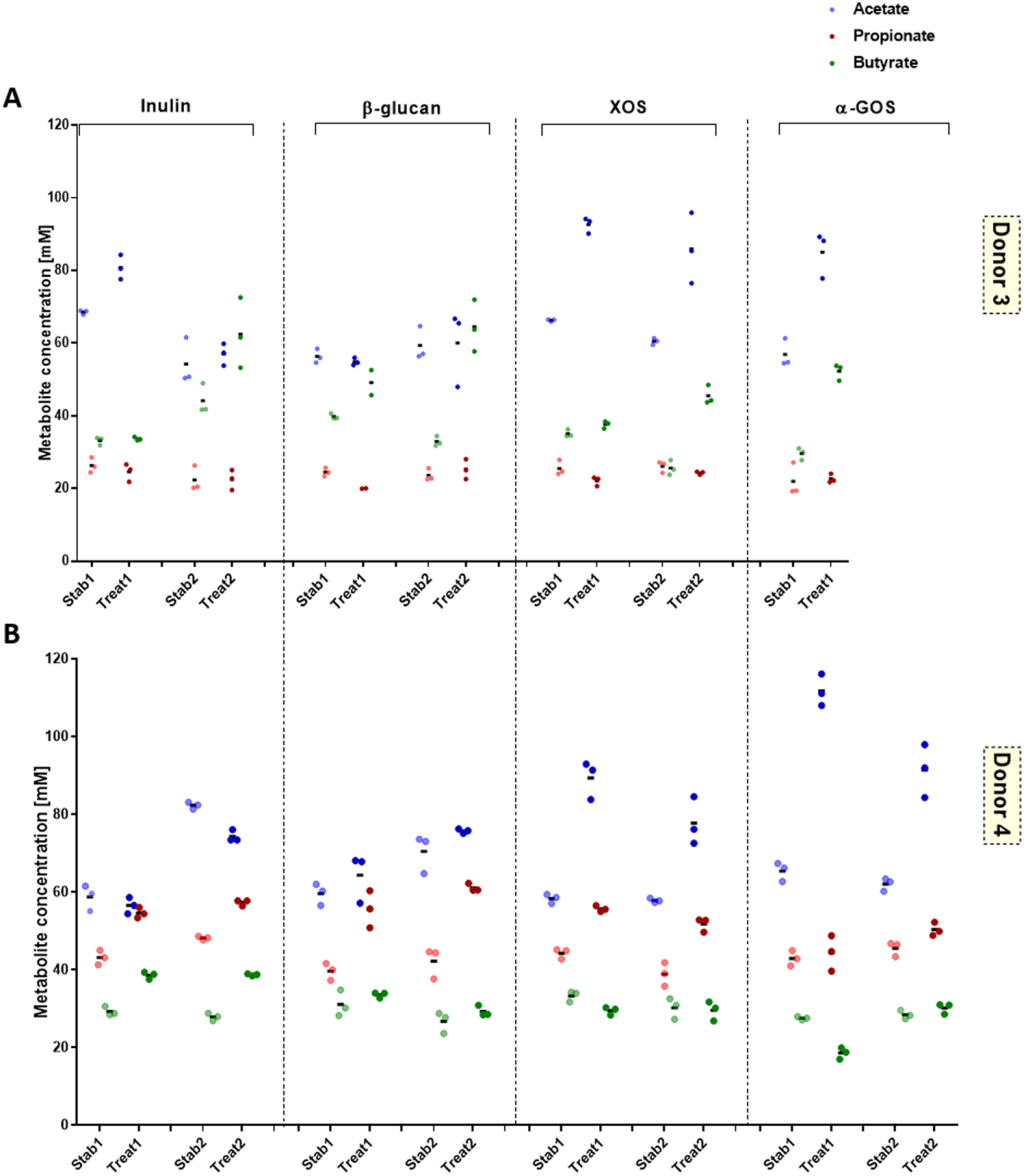
Effect of dietary fiber supplementation on fermentation metabolite concentration (**A** and **B**) and microbiota of fermentation 1 (F1) (donor 3) and fermentation 2 (F2) (donor 4). Mean (black horizontal line) from 3 consecutive measurements of acetate, propionate and butyrate concentrations (mM) in the respective reactor at end of stabilization and treatment phase for F1 (D3 microbiota) (**A**) and F2 (D4 microbiota) (**B**). Two replicates are shown for each dietary fiber treatment.

The effects of dietary fibers β-glucan, XOS, α-GOS and inulin on metabolic and microbial responses of stable PolyFermS microbiota of F1 and F2 were tested at a concentration of 4 g/L in the fermentation medium, mimicking an estimated daily intake of 9 g/day, for 7 days alternated by re-stabilization phases of 5 days, and two repetitions (noted I and II) were done for dietary fiber application within F1 and F2, except for α-GOS. Supplementation was performed through addition of sterile, non-heated fiber to the complex medium. Metabolic response of both microbiota was assessed by measuring SCFA production at the end of treatment (3 day sampling) and comparing to the production measured during the stabilization period before a fiber treatment is applied. Addition of dietary fibers yielded an overall increase in total SCFA production for both microbiota in F1 and F2 compared to stabilization, with mean increase ranging from 3 to 54 mM (F1) and 16 to 38 mM (F2), indicating fermentation of all supplemented fibers (Supplementary Figure S3). Acetate production was significantly enhanced for both microbiota upon supplementation with XOS and α-GOS, whereas during β-glucan treatment acetate levels remained stable. Butyrate and propionate productions were also increased by fiber supplementation but with microbiota-dependent response. Butyrate production increased (between 2 and 96%) in F1 (D3), while propionate production was enhanced (between 3 and 40%) in F2 (D4) microbiota upon β-glucan, XOS, α-GOS and inulin supplementation (Fig. 2A and B and Supplementary Table S6A). In addition, inulin also raised butyrate production in F2 (D4). Metabolic interactions within the PolyFermS microbiota upon fiber treatment were observed when inulin was supplemented. This is demonstrated by an increase in butyrate, when acetate levels remained stable or decreased (F1 treatment 2 and F2), but when acetate concentrations increased, butyrate levels remained stable (F1 treatment 1), suggesting cross-feeding of butyrate-producers on acetate.

**Figure 3:**
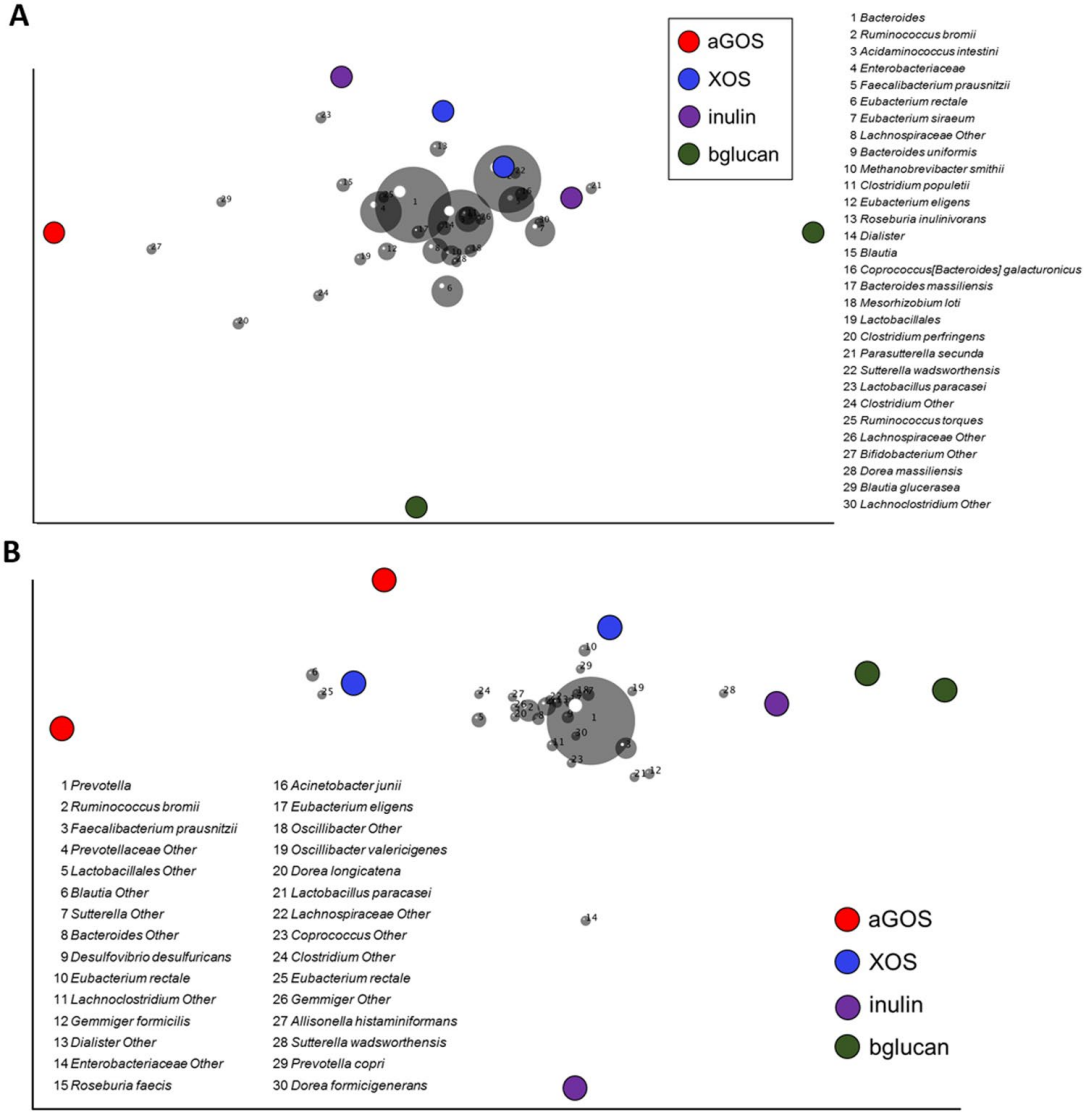
Effect of dietary fiber supplementation on microbiota (**A** and **B**) of fermentation 1 (F1; donor 3) and fermentation 2 (F2; donor 4). Principle components analysis (PCA) biplot showing variation among the PolyFermS microbiota of F1 (**B**) and F2 (**D**) after supplementation with dietary fibers XOS, a GOS, b glucan and inulin. Variables included in the PCA were relative abundance of OTUs (>0,05%) and OTUs are represented as numbers.

Interestingly we did not observe a consistent change of a single OTU upon fiber supplementation, confirming the current hypothesis of high functional redundancy within the gut microbiota and suggesting a multi-strain response causing observed changes in SCFA production. Principle coordinate analysis (PCoA) on weighted UniFrac distances of the fiber-supplemented PolyFermS microbiota allowed us to identify taxa that stratify the microbiota of F1 and F2 in relation to the dietary fiber supplementation and their abundances (Fig. 3A and B). Due to the different microbiota of F1 (*Bacteroidaceae*-*Ruminococcaceae* dominated) and F2 (*Prevotellaceae-Ruminococcaceae* dominated), the response on OTU level upon a dietary fiber was in some cases different. For example, α-GOS supplementation resulted in consistent higher levels compared to stabilization period of *Lachnospiraceae* in F2 microbiota during both treatment periods (I: 6 to 12% and II: 5 to 10%; Supplementary Table S7B), also reflected by a *Blautia* (6) and *Eubacterium rectale* (25) OTU in the PCoA. Whereas in F1 microbiota *Lachnospiraceae* levels decreased upon α-GOS supplementation (18 to 13%, Supplementary Table S7A). In PCoA, β-glucan supplemented F1 microbiota was determined by *Eubacteriaceae* OTUs (*E*. *siraeum* (7) and *E*. *rectale* (6)), in accordance with higher *Eubacteriaceae* levels compared to stabilization periods (I: 10 to 23% and II: 7 to 20%; Supplementary Table S7A). On the other hand, β-glucan supplemented F2 microbiota was separated in the PCoA by a *Sutterella wadsworthensis* OTU (28). An increased relative abundance of *Prevotellaceae* in both repetitions of β-glucan supplementation was also observed (I: 26 to 61% and II: 54 to 58%; Supplementary Table S7B) compared to the previous stabilization period. Interestingly, inulin showed consistent changes in relative abundance upon supplementation, independent of the donor microbiota. After inulin supplementation, the relative abundance of *Ruminococcaceae* increased in both fermentations (F1: 22 to 28% and F2: I: 13 to 19% and II: 16 to 22%; Supplementary Table S7B). No consistent changes in relative abundance of main bacterial families could be detected upon XOS supplementation in both microbiota (Supplementary Table 7). However, in the PCoA the XOS supplemented microbiota of F2 were both determined by an *E*. *rectale* OTU (10 and 25; Fig. 3B), which indicates a higher abundance of *E*. *rectale* in XOS supplemented F2 microbiota.

### Reproducible metabolic response to dietary fiber supplementation in PolyFermS model with same microbiota

Advances in research are based on the reproducibility of previously published data or investigated results and findings^35^. In this study, we performed a third fermentation with D3 microbiota 15 months after F1 with treatments inulin and α-GOS. In order to assess the microbial composition similarities or eventual differences after one year, the relative abundances (V4 region of 16S rRNA gene) of the different phyla and families were compared (Supplementary Table S8). At phylum level, fecal microbiota of donor D3 at both time points used to inoculate F1 and F3 was dominated by *Firmicutes* (F1 D3: 51% and F3 D3: 50%) and *Bacteroidetes* (F1 D3: 25% and F3 D3: 31%). The key bacterial families defining D3 microbiota remained stable with dominance of *Bacteroidaceae* (F1 D3: 17% and F3 D3: 22%) and *Ruminococcaceae* (F1 D3: 21% and F3 D3: 16%) and presence of *Verrucomicrobiaceae* (F1 D3: 6% and F3 D3: 4%) and *Methanobacteriaceae* (F1 D3: 9% and F3 D3: 6%).

**Figure 4:**
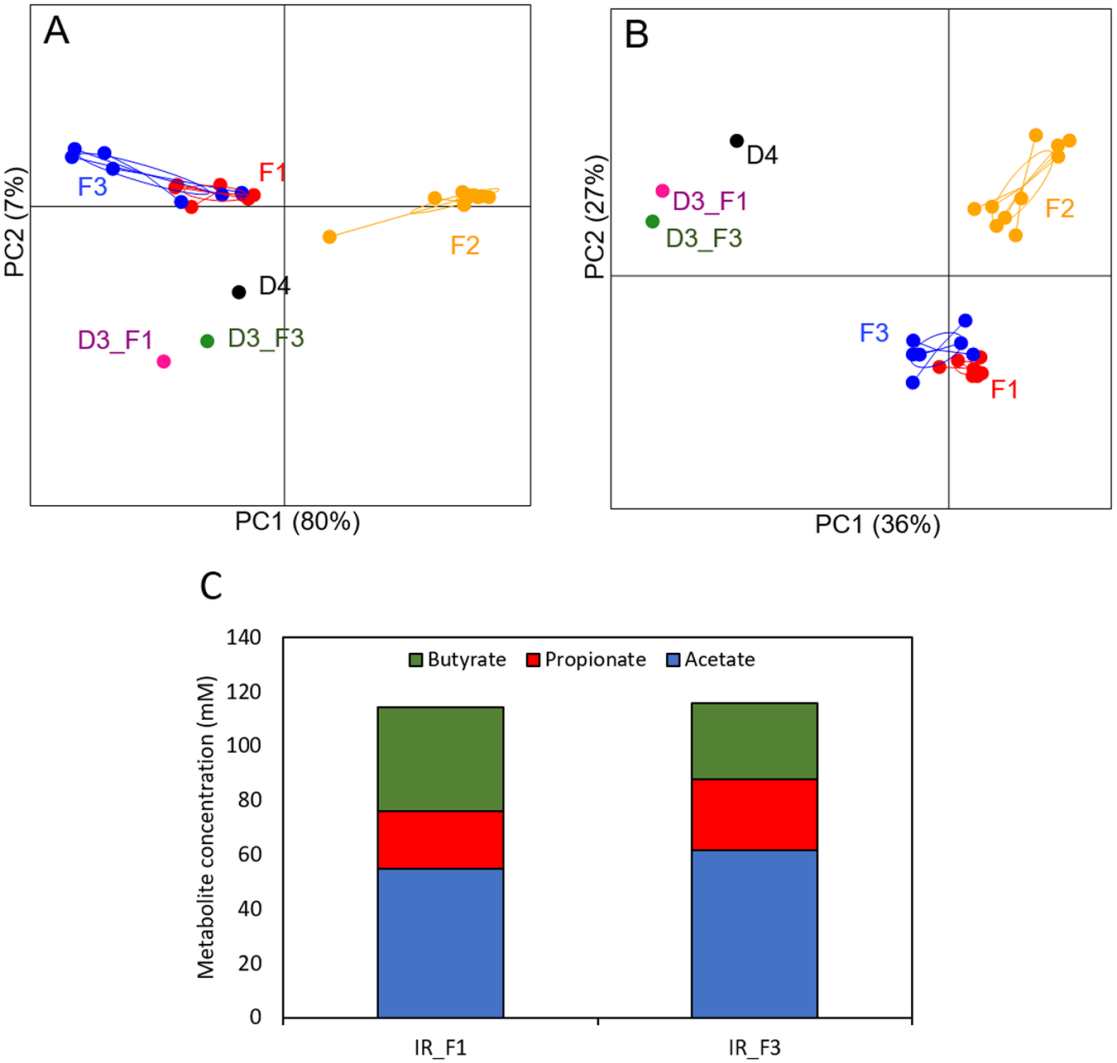
Reproducability over time of PolyFermS model inoculated with same healthy fecal donor (D3); F3 was operated 15 months after F1. PCoA plots of weighted (**A**) and unweighted (**B**) were performed based on the UniFrac distance matrix generated from sequencing V4 region of 16S rRNA genes in samples from donor’s feces and fermentation effluents of F1 (D3), F2 (D4) and F3 (D3). Each circle represents a sample from feces and effluent samples from F1 (D3) (red), F2 (D4) (orange) and F3 (D3) (purple). Bar-plot representation of the mean acetate (blue), propionate (red) and butyrate (green) concentrations (mM) (**C**) of PolyFermS effluent samples of inoculum reactor (IR) during stable operation phase and connection with experimental reactors (IR_F1: day 1–day 36; IR_F3: day 1–day 23); D3, donor 3; F1, fermentation 1; F2, fermentation 2; D4, donor 4; F3, fermentation 3.

In order to assess reproducibility and stability of the D3 microbiota in our PolyFermS model, the fermentation effluent microbiota of the non-treated reactors were compared to fecal donor and F1 and F2 microbiota. PCoA of UniFrac distances (Fig. 4A and B) showed F1 and F3 microbiota (D3) were more similar to each other on OTU composition (unweighted) and abundance (weighted) compared to F2 microbiota (D4), demonstrating the reproducible conservation of donor microbiota profile *in vitro*. The abundance of the dominant bacterial families was also comparable between F3 and F1 microbiota in inoculum reactors with *Bacteroidaceae* (F3: 29%; F1: 26%) and *Ruminococcaceae* (F3: 19%; F1: 19%) as predominant families (Supplementary Table S8B).Total SCFA production was also comparable between F3 and F1 with 129 ± 7 mM and 123 ± 10 mM, respectively (Fig. 4C). There was a slight difference in SCFA profile in the IR effluents, with lower butyrate levels in F3 (22%) compared to F1 (31%) but comparable acetate (F3: 48%; F1: 45%) and propionate (F3: 20%; F1: 17%) concentrations (Fig. 4C).

**Figure 5:**
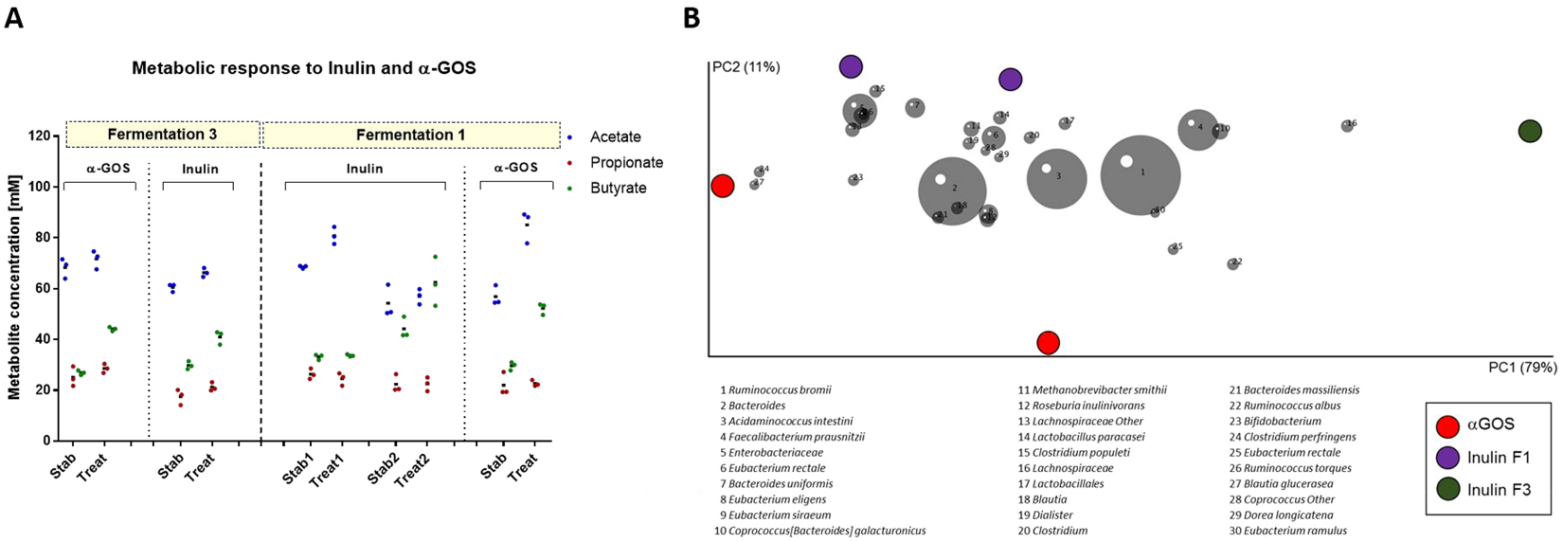
Repetition of α-GOS and inulin supplementation to D3 microbiota in PolyFermS resulted in comparable higher butyrate productions and stimulates specific OTUs (PCA). (**A)** Mean (black horizontal line) from 3 consecutive measurements of acetate, propionate and butyrate concentrations (mM) in the respective reactor at end of stabilization and treatment phase for fermentation 1(F1) and fermentation 3 (F3) (donor 3).Two replicates are shown for each dietary fiber treatment. (**B**) Principle components analysis (PCA) biplot showing variation in the PolyFermS microbiota of F1 and F3 after supplementation with dietary fibers a GOS and inulin. Variables included in the PCA were relative abundance of OTUs (>0,05%) and OTUs are represented as numbers.

After reaching stable SCFA profile in reactors during stabilization, supplementation with inulin or α-GOS resulted in significant increases of total SCFAs (Fig. 5A**;** Supplementary Table S6B). Similar to F1, addition of α-GOS (from 28 ± 2 to 44 ± 1 mM, during stabilization and treatment, respectively) or inulin (from 30 ± 2 to 41 ± 3 mM) enhanced butyrate production, while inulin stimulated acetate production (from 61 ± 2 to 66 ± 2 mM). α-GOS supplementation resulted in increased levels of *Ruminococcaceae* (from 24 to 31% during stabilization and treatment, respectively) and *Bacteroidaceae* (from 22 to 26%), (Supplementary Table S9). Simultaneously a decrease in *Eubacteriaceae* (17 to 10%) was observed. The relative abundance of *Ruminococcaceae* was increased by inulin supplementation (from 41 to 48%), while abundance of the *Eubacteriaceae* decreased (from 15 to 9%).

## Discussion

In this study the effect of supplementation of four different dietary fibers on human gut microbiota was investigated at the levels of metabolic and bacterial composition using a continuous *in vitro* fermentation system, modeling adult proximal colon conditions. By using two distinct fecal microbiota composition we could demonstrate a consistent and donor-dependent butyrogenic or propionigenic response towards the fiber treatments.

Both on metabolic and phylogenetic levels we successfully maintained two distinct colon microbial communities in the PolyFermS reactors reflecting the corresponding fecal microbiota donor. Both fecal microbiota were dominated by Firmicutes and Bacteroidetes species and differed mainly on family level within the Bacteroidetes phylum with higher levels of *Bacteroidaceae* species in D3, low in D4, and high levels of *Prevotellaceae* in D4, low in D3. Several other reports observed that individuals with high levels of *Prevotella* have low levels of *Bacteroides* and vice versa^34,36,37^, suggesting niche competition within the human gut microbiota. Abundance of *Bacteroides* has been associated with animal/fat-rich diets^36,37^, while high *Prevotella* abundance has been associated with plant-rich and vegetarian diets^36,38^. Difference in abundance of Bacteroidetes phylum between the two donor microbiota was preserved and even enhanced in our PolyFermS model for proximal colon microbiota, despite identical chime-simulating fermentation medium composition. The PolyFermS abundance levels fall within the range reported in a recently published large cohort of 1106 fecal microbiota of Western European individuals with *Bacteroides*: 0.1–72% and *Prevotella*: 0–56%^39^. Stable SCFA profiles were obtained in the untreated control reactors of F1 and F2, reflecting maintenance of functional stability of bacterial community during continuous operation of 36 and 43 days, respectively. The *Prevotellaceae*-dominated microbiota (F2) was characterized by high propionate production and can be explained by propionate –producing capacity of *Prevotella* species^40^. It was earlier reported that higher fecal propionate levels are associated with *Prevotella* species^41,42^. In another cohort it was shown that individuals with more than 20% *Prevotella* have higher levels of methylmalonyl-CoA mutase, a key enzyme involved in propionate production, in their fecal metaproteome^43^. The high butyrate production in F1 (*Bacteroidaceae*-dominated) microbiota is likely due butyrate-producing *Lachnospiraceae* and *Ruminococcaceae* species, which were the second and third dominating families within F1. *Bacteroidaceae* species do not produce butyrate within the human gut^40,44^, but may contribute to the butyrate pool by their acetate production, used as co-substrate during butyryl-CoA-transferase route in gut bacteria^45^.

Our data showed that all dietary fibers supplemented in the PolyFermS microbiota increased SCFA production, displaying fermentability of all tested substrates by both microbiota. Overall, F1 microbiota responded to all dietary fibers by increased butyrate production, whereas F2 microbiota showed increased propionate production. This specific response was consistent among the different treatments and the three fermentations. Similar inter-individual differences in butyrogenic or propionigenic response were observed in static batch experiments with fecal microbiota supplemented with wheat bran particles or inulin^46^, and with FOS and two arabinoxylan variants^47^. Comparable to our observations, Chen *et al*.^47^ showed that *Prevotella*-dominated microbiota responded by higher propionate production upon fiber supplementation *in vitro*. Both in F1 and F2, XOS and αGOS resulted in strong increase in acetate levels, which is produced by almost all heterotrophic gut bacteria^48^. Both dietary fibers were short-chain types, which makes them easier fermentable^49^ and it was shown *in vitro* that various intestinal bacteria can use GOS and XOS^50^. This broad-range utilization may explain the different effects measured on microbiota composition upon XOS supplementation. Metabolic cross-feeding between acetate- and butyrate- producers resulting in higher butyrate levels was observed with inulin in both PolyFermS microbiota, and repetitions. Inulin can be degraded by different *Bifidobacterium* spp., *Lactobacillus* spp.^51^ and some butyrate-producing *Roseburia* spp.^52^, *F*. *prausnitzii* and *Eubacterium rectale*^53^. Bifidobacteria and lactobacilli produce acetate and lactate, which can in turn be utilized by butyrate-producing bacteria. During these cross-feeding interactions on inulin-type fructans both commensalism (cross-feeding on acetate and lactate) and substrate competition occurs between both bacterial groups in co-culture experiments^54^. We observed a consistent increase in *Ruminococcaceae*, but no increase of *Actinobacteriaceae* (bifidobacteria), which suggests that in our set-up the inulin-degrading *Ruminococcaceae* (e. g. *F*. *prausnitzii*) produced butyrate while consuming the available acetate in the mixed microbial environment. Indeed, increased *Ruminococcaceae* levels upon inulin treatment was linked with an increase in a *F*. *prausnitzii* OTU, which is in accordance with *in vitro*^55^ and human observations^56,57^. Β-glucan supplementation resulted in increased butyrate and *Eubacteriaceae* levels in F1 microbiota, and increased propionate and *Prevotellaceae* levels in F2. Both changes in microbial composition explain the change in metabolic profile as *Eubacteriaceae* species produce butyrate and acetate, which becomes available for cross-feeding interactions, while *Prevotellaceae* produce propionate^40^. The increase in *Bacteroides-Prevotella* group and propionate production was also observed *in vitro* with oat β-glucan^58^. *Prevotella* can better ferment complex polysaccharides from the diet than *Bacteroides*^59^, which may explain their competitive advantage upon β-glucan supplementation.

Overall, we did not detect consistent or systematic changes in the microbiota composition upon dietary fiber supplementation. It appears that the microbiota modulation by dietary fibers occurs at species level as demonstrated by Chung *et al*.^55^ in a continuous *in vitro* fermentation with three different fecal microbiota and dietary fibers as sole carbohydrate source. Due to the higher inter-individual variation at species level within the human gut microbiota, it can be expected that a single fiber will not induce a strong specific modification of the microbiota at species level in different individuals. Indeed, human intervention studies with dietary fibers showed marked inter-individual microbiota changes, which were depended on the individuals’ dominant microbiota composition^9^.

Compared with *in vivo* and human studies, there are limitations of the *in vitro* approach, since the models do not replicate all the conditions that occur in the colon, resulting in enriching and diminishing of bacterial populations. However, our data correspond well with dietary fiber fermentation *in vivo* and allowed insight into the complex cross-feeding mechanisms. In particular, we managed to show that dietary fibers induce dynamic responses depending on an individual’s specific microbiota. Main strength of *in vitro* fermentation models for prebiotic research is that one can follow the *in situ* SCFA production upon treatment, whereas in human intervention studies fecal SCFA concentrations are only a proxy for colonic fermentation and mainly a result of absorption of the SCFA in the intestine and lack thereof. Our approach of repeated dietary fiber supplementation to a stable and active gut microbiota in a continuous fermentation model allowed elucidating direct and indirect metabolic and compositional shifts that can occur in the human gut using long-term supplementation. This is in contrast to often used static batch incubations with fecal microbiota and dietary fibers, which reflect a direct effect of a fiber on the growth and activity of fast substrate utilizers and often neglects the indirect effects or long term shifts that might have the most profound effect on microbiome composition.

To summarize, our study showed that two distinct fecal microbial consortia maintained *in vitro* in the PolyFermS continuous intestinal fermentation model inoculated with immobilized adult fecal microbiota responded differently to dietary fiber supplementation on metabolic and compositional level. Irrespective to the dietary fiber supplemented. The *Bacteroidaceae-Ruminococcaceae* dominated microbiota produced more butyrate while the *Prevotellaceae-Ruminococcaceae* dominated microbiota produced more propionate. No fiber-specific change on phylogenetic level was observed, but changes in abundance of specific families or species level OTUs within a microbiota together with cross-feeding interactions between the different functional groups could explain the observed changes in SCFA profiles. Our data suggest that the metabolic profile of SCFA may be the most suitable and robust read-out to characterize the microbiota-modulating effect of a fiber and emphasize on the importance to understand inter-individual responses to a prebiotic treatment for mechanistic understanding and human application.

## Material and Methods

### Fecal bacteria immobilization

Fecal samples were donated by two healthy individuals (male, age 33 and 32), who did not receive antibiotic or probiotic supplementation for at least 3 months before donation. Fecal samples were collected in a sterile 50 mL Falcon tube in an airtight container together with one Anaerogen sachet (Oxoid) to obtain anaerobic conditions until transfer into an anaerobic chamber (10% CO_2_, 5% H_2_ and 85% N_2_) within 3 h (Coy Laboratories, Ann Arbor, MI, USA). Fecal bacteria were immobilized in 1–2 mm gel beads consisting of gellan gum (2.5%, w/v), xanthan (0.25%, w/v) and sodium citrate (0.2%, w/v) under anaerobic conditions as previously described in detail^30,32^. Sixty mL of freshly produced fecal beads were transferred in the IR bioreactor containing 140 mL of nutritive medium. For bead colonization, three consecutive fed-batch fermentations were carried out by replacing 100 mL fresh nutritive medium every 8–12 h. Bacteria, growing close to the bead surface are continuously released into the growth medium due to active cell growth in the high-biomass-density peripheral layer^29,60^.

### Nutritive medium

The nutritive medium was based on the composition described by Macfarlane *et al*.^61^ for simulation of the chyme in the adult human colon. It included (g L^−1^ of distilled water): pectin (citrus) (2), xylan (oat spelts) (2), arabinogalactan (larch) (2), guar gum (1), inulin (1), soluble potato starch (5), mucine (4), casein acid hydrolysate (3), peptone water (5), tryptone (5), yeast extract (4.5), L-cysteine HCl (0.8), bile salts (0.4), KH_2_PO_4_ (0.5), NaHCO_3_ (1.5), NaCl (4.5), KCl (4.5), MgSO_4_ anhydrated (0.61), CaCl_2_*2 H_2_O (0.1), MnCl_2_* 4 H_2_O (0.2), FeSO_4_* 7H_2_0 (0.005), hemin (0.05) and Tween 80 (1 mL). Prior sterilization (20 min, 120 °C), the pH of the medium was adjusted to 5.7. One mL of a filter-sterilized (0.2 μm pore-size) vitamin solution^62^ was added to the sterilized and cooled down medium. All components of the fermentation medium were purchased from Sigma-Aldrich Chemie (Buchs, Switzerland), except for peptone water (Oxoid AG, Pratteln, Switzerland), inulin (Orafti®, RPN Food-technology AG, Sursee, Switzerland), bile salts (Oxoid AG), tryptone (Becton Dickinson AG, Allschwill, Switzerland), yeast extract (Merck, Darmstadt, Germany), KH_2_PO_4_ (VWR International AG), NaHCO_3_ (Fluka, Buchs, Switzerland), NaCl (VWR international AG, Dietikon, Switzerland), KCl (Fluka, Buchs, Switzerland) and KH_2_PO_4_ (VWR International AG).

Four different dietary fibers (inulin-type fructan, β-glucan, XOS, α-GOS) were investigated (Supplementary Table S1) and supplemented to sterile nutritive medium at a concentration of 4 g L^−1^, calculated for an estimated daily intake of 9 g L^−1^, accounting for the reactor volume of 0.2 L compared to 0.75 L for the proximal colon volume, and a chime medium supply of 0.6 L medium per day, giving a mean retention time of 8 h. Complete hydration of dietary fibers was allowed for 24 h under high speed stirring at 4 °C, as presented below.

### Simulation of proximal colon microbiota and treatment with dietary fibers

#### Experimental set-up

**Figure 6:**
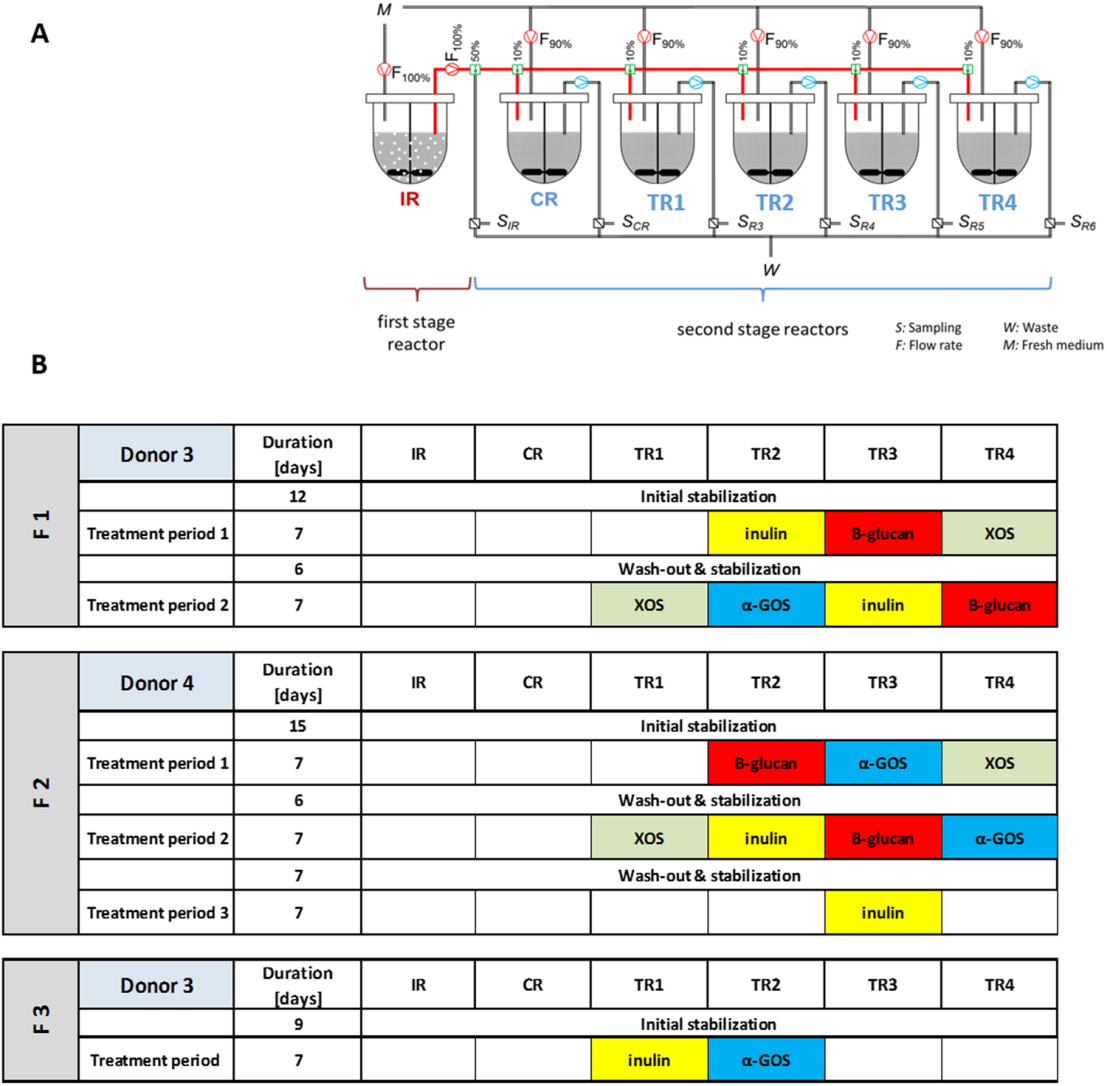
Experimental set-up (**A**) and time schedule (**B**) of the continuous fermentation model with inoculum reactor (IR) and control (CR) and treatment (TR) reactors. CR and TR’s were fed with effluent from IR and with nutritive medium during stabilization and washout periods. TRs were fed with effluent from IR and supplemented fermentation medium (4 g/L dietary fiber) during treatment periods.

The set-up of the PolyFermS model was adapted for the aim of the study and is schematized in Fig. 6A. The continuous fermentations were carried out for up to 42 days in a two-stage design with a total of six bioreactors (Sixfors, Infors, Bottmingen, Switzerland). Each reactor was operated with conditions selected to mimic the adult proximal colon (pH 5.7, stirring at 120 rpm, 37 °C, and mean retention time of 8 h). Anaerobiosis was maintained through continuous headspace flushing with CO_2_, and a constant pH of 5.7 was maintained by addition of 2.5 M NaOH. IR had an operation volume of 200 mL and was inoculated with 60 mL of fecal beads and connected via a peristaltic pump (Reglo, Ismatec, Glattbrugg, Switzerland) to all second stage reactors, one control reactor (CR) and four treatment reactors (TR1-4) operated in parallel. All reactors were operated at a working volume of 200 mL. Fresh sterile nutritive medium was continuously supplied to IR at a flow rate of 25 mL/h, and second stage reactors were inoculated with 5% (v/v) (1.25 mL/h) IR effluent and supplied with 95% (v/v) (23.75 mL/h) fresh fermentation medium.

#### Experimental procedure

An initial colonization and stabilization phase of up to 15 days, prior experimentation was done (Fig. 6B). Each treatment was performed for 7 days for reaching a stable state, monitored by metabolite analysis and base consumption. The treatment periods were alternated with re-stabilization phases of 5–7 days, aiming to washout effects of the previous applied treatment and re-establishment of a microbiota composition similar to that in IR. The DFs (Supplementary Table S1) were added to the medium (4 g/L) and connected to assigned treatment reactor.

Effluent samples were taken daily and separated into bacterial pellet (10 min of centrifugation at 14.000 g at 4 °C) and supernatant, and stored at −20 °C until further analysis. Stability of the reactor microbial communities was monitored by daily measurements of main fermentation metabolites concentrations in sample supernatant. Standard observed variations in the PolyFermS fermentation metabolites are normally lower than 10% and used to define functional microbial stability, before starting and analyzing samples of a treatment period.

### Microbial metabolite analysis

High performance liquid chromatography (HPLC) analysis (Thermo Fisher Scientific Inc. Accela, Wohlen, Switzerland) was performed to determine the concentrations of SCFAs (acetate, butyrate and propionate), branched-chain fatty acids (BCFAs) (isobutyrate, valerate and isovalerate) and intermediate metabolites (lactate and formate) produced by the microbiota in the reactor effluents. Analyses were performed with a Hitachi LaChrome device (Merck, Dietikon, Switzerland) using a Cation-H refill cartridge (30 × 4.6 mm) connected to an Aminex® HPX-87H (300 × 7.8 mm) column. Due to biofilm formation, supernatants of IR and TR reactors were diluted in ultrapure water and filtered through a 0.22 or 0.45 μm nylon membrane (Infochroma AG, Zug, Switzerland) into glass vials and sealed with crimp-caps. Around 40 μL of the sample were injected into the HPLC with a flow rate of 0.4 mL min^−1^ and H_2_SO_4_ as an eluent.

### Microbial community analysis

#### Genomic DNA extraction

The genomic DNA was extracted from 200 mg feces and pellet of 2 mL PolyFermS effluent using the FastDNA® SPIN Kit for Soil (MP Biomedicals, Illkirch, France). Total DNA concentration (ng/μL) and purity was determined by spectrophotometry using Nanodrop (Nanodrop ND 1000 Spectrophotometer, Thermo Scientific, Wilmington, USA).

#### Microbiota profiling with 16S rRNA gene amplicon sequencing

The bacterial composition of the fecal and PolyFermS samples was determined using tag-encoded 16S rRNA gene Miseq-based (Illumina, CA, USA) high throughput sequencing. DNA samples of the last day of each experimental period of each PolyFermS reactor were selected for assessing the bacterial composition and its stability in the PolyFermS model and the shifts after prebiotic treatments. The V4 region of the 16S rRNA gene was amplified with modified primers 515 F (TATGGTAATTGTGTGNCAGCMGCCGCGGTAA) and 806 R (AGTCAGTCAGCCGGACTACHVGGGTWTCTAAT). Library preparation and sequencing was performed by StarSEQ (Mainz, Germany) using for sequencing one MiSeq cell and the V2 2 × 250 bp paired end Next Tera chemistry supplemented with 20% of PhiX.

The raw data set containing pair-ended reads with corresponding quality scores were merged using settings as previously mentioned^63^. The minimum length of merged reads was 200 bp. Quantitative Insight Into Microbial Ecology (QIIME) open source software package (1.7.0, 1.8.0 and 1.9.0) was used for subsequent analysis steps^64^. Purging the dataset from chimeric reads and constructing de novo Operational Taxonomic Units (OUT) was conducted using the UPARSE pipeline^65^. The custom human intestinal 16S rRNA database (HITdb) was used as a reference database^66^. Alpha and beta diversity analysis was performed as previously described using iterative subsampling (36,000 reads/sample)^67^.

### Statistical analysis

All statistical analysis for HPLC were carried out using the SigmaPlot 13.0 version, San Jose, California, USA. HPLC Data are expressed as means ± Standard deviations (SD) of three days at the end of the stabilization and treatment period of each fermentation. HPLC data were compared between stabilization and treatment phase using the nonparametric Shapiro-Wilk test. *P*-values < 0.05 were considered significant. Brown-Forsythe test was used to determine differences between stabilization and treatment periods.

## Electronic supplementary material

Supplementary Figures and Tables
