# Supplementary material for: Understanding the prebiotic potential of different dietary fibers using an in vitro continuous adult fermentation model (PolyFermS)
Source: Sci Rep. 2018 Mar 12;8:4318. doi: 10.1038/s41598-018-22438-y (PMC5847601; doi:10.1038/s41598-018-22438-y)
Supplement: Supplementary file 1 — Supplementary Figures and Tables [file 41598_2018_22438_MOESM1_ESM.pdf]

Understanding the prebiotic potential of different dietary fibers using an *in vitro* continuous adult fermentation model (PolyFermS)

Sophie A. Poeker[1], Annelies Geirnaert[1], Laura Berchtold[1], Anna Greppi[1], Lukasz Krych[2], Robert E. Steinert[3], Tomas de Wouters[1] and Christophe Lacroix[1*]

Dietary fiber	Trade name, Supplier	Full name	Sugar moieties	Linkage	Degree of polymerization	Structure	Source	Purity
Inulin-type fructan	Fibrulose F97<br>(Cosucra Group, Warcoing, Belgium)	Fructo-oligosaccharide	fructose, glucose	*β*-(2,1)<br><br>terminal Glc: *α*–(1,2)	2-20	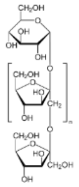	chicory roots	DM: 96 ± 1 %<br>DP≤ 10: 70 ± 5% on DM<br>DP≤ 20: 94 ± 5% on DM<br>DP >20: 5 ± 5% on DM
α-GOS	Cravingz'Gone® P<br>(Olygose, Compiégne Cedex, France)	*α*-galacto-oligosaccharide	galactose, glucose	*α*–(1,6)	2 (melibiose)<br>3 (manninotriose)	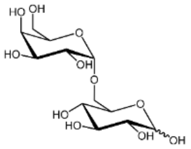	peas	99%
β-glucan	Beta Glucan Powder (CEPARO, Edmonton, Canada)	*β*-(1,3)-(1,4)-D-glucan	glucose	*β*-(1,3)<br>*β*-(1,4)	fiber	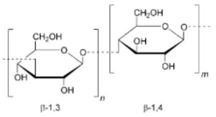	oats	90%
XOS	XOS 95P<br>(Longlive, Shandong, China)	Xylo-oligosaccharide	D-xylose	*β*-(1,4)	2-7	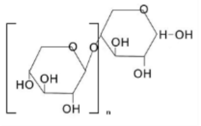	ligno-cellulosic wastes	≥95% DP 2-7

Supplementary Table S1: Characteristics of the dietary fibers used for medium supplementation during treatment periods.

A

Phylum	D3	D4
Firmicutes	51%	57%
Bacteroidetes	25%	32%
Euryarchaeota	9%	2%
Actinobacteria	6%	6%
Verrucomicrobia	6%	-
Proteobacteria	3%	1%
Tenericutes	-	3%

B

Phylum	Family	D3	D4
Firmicutes	*Ruminococcaceae*	20.9%	19.7%
Firmicutes	*Lachnospiraceae*	7.3%	13.4%
Bacteroidetes	*Bacteroidaceae*	16.9%	8.4%
Bacteroidetes	*Prevotellaceae*	0.1%	17.2%
Euryarchaeota	*Methanobacteriaceae*	8.7%	1.6%
Firmicutes	*Oscillospiraceae*	6.8%	4.0%
Bacteroidetes	*Rikenellaceae*	6.0%	3.4%
Verrucomicrobia	*Verrucomicrobiaceae*	6.2%	0.0%
Firmicutes	Unclassified Clostridiales	2.7%	5.3%
Firmicutes	*Eubacteriaceae*	4.3%	7.1%
Actinobacteria	*Bifidobacteriaceae*	3.6%	4.1%
Firmicutes	*Clostridiaceae*	2.9%	1.5%
Firmicutes	unclassified	0.2%	2.4%
Proteobacteria	RF32; unclassified	0.1%	-
Bacteroidetes	*Porphyromonadaceae*	2.3%	3.6%
Firmicutes	*Peptostreptococcaceae*	2.2%	1.2%
Firmicutes	*Erysipelotrichaceae*	0.8%	2.1%
Proteobacteria	*Desulfovibrionaceae*	2.0%	0.2%
Bacteroidetes	*Odoribacteriaceae*	-	-
Actinobacteria	*Coriobacteriaceae*	1.5%	1.3%
Bacteroidetes	*Paraprevotellaceae*	-	-
Firmicutes	*Acidaminococcaceae*	0.3%	-
Firmicutes	*Christenellaceae*	0.9%	0.3%
Firmicutes	*Erysipelotrichaceae*	0.8%	2.1%
Firmicutes	*Veillonellaceae*	0.7%	0.6%
	Sum	98.2%	99.5%

Supplementary Table S2: Taxonomic summary of fecal microbiota of selected healthy human donors for PolyFermS experiments. Abundance of phyla (**Table 2. A**) and families (**Table 2. B**) obtained by 16S amplicon sequencing of V4 region.

Shannon index					
Fermentation 1		Fermentation 2		Fermentation 3	
D3	5.77 ± 0.92	D4	5.82 ± 0.89	D3	**3.84 ± 0.39**
IR_D12	4.52 ± 0.01	IR_D8	3.58 ± 0.41	IR_D8	**4.07 ± 0.01**
IR_D19	4.33 ± 0.52	IR_D14	3.35 ± 0.38	IR_D14	**4.02 ± 0.01**
IR_D24	4.39 ± 0.51	IR_D19	3.37 ± 0.31	CR_D8	**3.89 ± 0.43**
TR_D31	4.32 ± 0.54	IR_D25	3.37 ± 0.36	CR_D14	**3.83 ± 0.39**
CR_D12	4.08 ± 0.53	IR_D31	3.91 ± 0.41		
CR_D19	4.30 ± 0.45	IR_D37	3.86 ± 0.49		
CR_D24	4.15 ± 0.47	CR_D8	3.61 ± 0.39		
CR_D31	4.35 ± 0.52	CR_D14	3.60 ± 0.39		
		CR_D14	3.49 ± 0.39		
		CR_D25	3.34 ± 0.40		
		CR_D31	3.77 ± 0.37		
		CR_D37	3.73 ± 0.42		

Supplementary Table S3: Mean Shannon diversity index of inoculum reactor (IR) and control reactor (CR) samples for different time points along the fermentation periods. Data shown are means ± SD obtained of 11 replicates per sample.

Phylum	Fermentation 1							
	IR_d12	IR_d19	IR_d24	IR_d31	CR_d12	CR_d19	CR_d24	CR_d31
Firmicutes	58%	56%	54%	62%	65%	55%	42%	57%
Bacteroidetes	27%	25%	22%	16%	24%	28%	37%	28%
Proteobacteria	14%	18%	22%	15%	11%	15%	21%	13%
Euryarchaeota	1%	1%	1%	2%	0%	2%	0%	2%
Actinobacteria	0%	0%	0%	5%	0%	0%	0%	0%
Verrucomicrobia	0%	0%	0%	0%	0%	0%	0%	0%

Phylum	Fermentation 2											
	IR_d8	IR_d14	IR_d19	IR_d25	IR_d31	IR_d37	CR_d8	CR_d14	CR_d19	CR_d25	CR_d31	CR_d37
Bacteroidetes	56%	60%	60%	61%	50%	52%	61%	62%	62%	62%	52%	56%
Firmicutes	40%	34%	34%	35%	41%	33%	35%	33%	32%	34%	40%	36%
Proteobacteria	4%	6%	6%	3%	9%	14%	4%	5%	5%	3%	7%	7%
Actinobacteria	0%	0%	0%	0%	0%	0%	0%	0%	0%	0%	0%	0%
Euryarchaeota	0%	0%	0%	0%	0%	0%	0%	0%	0%	0%	0%	0%
Verrucomicrobia	0%	0%	0%	0%	0%	0%	0%	0%	0%	0%	0%	0%

Supplementary Table S4: Summary microbial phyla obtained by V4 region 16S amplicon sequencing within non-treated PolyFermS reactors. Inoculum reactor (IR) and control reactor (CR) with donor 3 microbiota (Fermentation 1) on day 12, day 19, day 24 and day 31. Inoculum reactor (IR) and control reactor (CR) with donor 4 microbiota (Fermentation 2) on day 8, day 14, day 19, day 25, day 31 and day 37.

Phylum	Family	Fermentation 1							
		IR_d12	IR_d19	IR_d24	IR_d31	CR_d12	CR_d19	CR_d24	CR_d31
Bacteroidetes	*Bacteroidaceae*	27.0%	25.4%	22.3%	16.2%	23.4%	28.3%	37.1%	27.7%
Firmicutes	*Lachnospiraceae*	13.4%	13.1%	12.0%	8.4%	14.0%	14.8%	9.8%	19.3%
Firmicutes	*Acidaminococcaceae*	12.6%	10.5%	11.6%	11.2%	15.1%	11.8%	10.4%	11.5%
Firmicutes	*Ruminococcaceae*	18.4%	19.4%	13.8%	21.8%	20.7%	6.7%	5.6%	11.4%
Proteobacteria	*Enterobacteriaceae*	9.6%	14.9%	19.6%	14.4%	5.5%	8.8%	14.0%	9.9%
Firmicutes	*Eubacteriaceae*	8.6%	7.6%	10.5%	15.1%	4.9%	16.3%	12.7%	8.2%

Phylum	Family	Fermentation 2											
		IR_d8	IR_d14	IR_d19	IR_d25	IR_d31	IR_d37	CR_d8	CR_d14	CR_d19	CR_d25	CR_d31	CR_d37
Bacteroidetes	*Prevotellaceae*	52.8%	58.4%	58.2%	58.1%	44.3%	48.6%	50.6%	52.7%	57.9%	58.2%	48.4%	52.3%
Firmicutes	*Ruminococcaceae*	19.7%	19.6%	18.0%	18.9%	25.0%	14.0%	17.1%	17.0%	14.3%	20.1%	21.0%	18.9%
Firmicutes	*Lachnospiraceae*	8.5%	5.9%	6.8%	8.1%	6.6%	9.8%	8.0%	7.3%	5.5%	5.9%	5.5%	6.8%
Firmicutes	unclassified Lactobacillales	3.6%	2.9%	3.8%	3.3%	4.0%	3.7%	3.6%	3.5%	7.1%	4.0%	7.1%	4.4%
Bacteroidetes	*Bacteroidaceae*	2.6%	1.8%	1.8%	2.5%	4.8%	2.5%	10.1%	9.2%	3.7%	3.4%	3.6%	2.8%

Supplementary Table S5: Summary of most abundant (>5%) microbial families obtained by 16S V4 region amplicon sequencing within non-treated PolyFermS reactors. Inoculum reactor (IR) and control reactor (CR) with donor 3 microbiota (Fermentation 1) on day 12, day 19, day 24 and day 31. Inoculum reactor (IR) and control reactor (CR) with donor 4 microbiota (Fermentation 2) on day 8, day 14, day 19, day 25, day 31 and day 37.

A

Ferm	Total SCFAs	Inulin	b-glucan	XOS	α-GOS
1	Stabilization I	138.54 ± 3.35	131.24 ± 2.84	136.82 ± 2.75	113.93 ± 6.39
	Treatment I	143.68 ± 6.11	133.91 ± 6.53	161.64 ± 7.13	167.79 ± 5.54[c]
	Stabilization II	125.91 ± 13.84	120.94 ± 7.93	116.25 ± 4.96	
	Treatment II	150.11 ± 6.35	157.71 ± 20.83	162.35 ± 12.09[b]	
2	Stabilization I	133.67 ± 7.62	138.87 ± 10.12	133.77 ± 3.63	142.38 ± 2.83
	Treatment I	154.40 ± 2.84[a]	154.83 ± 12.85	160.48 ± 5.29[b]	175.48 ± 9.37[b]
	Stabilization II	166.89 ± 3.17	145.75 ± 14.44	139.69 ± 7.05	138.92 ± 6.81
	Treatment II	175.84 ± 2.45[a]	172.62 ± 1.88[a]	178.03 ± 3.56[b]	174.76 ± 3.89[b]
	**Acetate**	**Inulin**	**b-glucan**	**XOS**	**α-GOS**
1	Stabilization I	68.56 ± 0.59	56.37 ± 1.96	56.37 ± 1.76	56.88 ± 3.88
	Treatment I	80.82 ± 3.37[b]	54.90 ± 1.01	92.62 ± 1.76[b]	85.10 ± 6.30[b]
	Stabilization II	54.26 ± 6.38	59.41 ± 4.60	66.32 ± 0.28	
	Treatment II	57.02 ± 3.01	60.08 ± 10.48	92.62 ± 2.15[c]	
2	Stabilization I	58.76 ± 3.31	59.63 ± 2.77	57.88 ± 0.55	65.44 ± 2.44
	Treatment I	56.56 ± 2.11	64.38 ± 6.23	77.78 ± 6.14[b]	111.78 ± 4.10[c]
	Stabilization II	82.31 ± 0.87	70.48 ± 4.94	58.38 ± 1.19	62.11 ± 1.67
	Treatment II	74.32 ± 1.52[a]	75.74 ± 0.58	89.39 ± 4.87[c]	91.43 ± 6.82[b]
	**Propionate**	**Inulin**	**b-glucan**	**XOS**	**α-GOS**
1	Stabilization I	26.36 ± 2.10	24.51 ± 1.17	24.51 ± 0.93	21.96 ± 4.53
	Treatment I	24.57 ± 2.47	18.78 ± 2.16[a]	22.10 ± 0.22	22.70 ± 1.20
	Stabilization II	22.37 ± 3.47	42.22 ± 3.95	25.56 ± 2.02	
	Treatment II	22.47 ± 2.73	61.12 ± 1.00[b]	22.10 ± 1.27	
2	Stabilization I	43.17 ± 1.88	39.66 ± 2.18	38.92 ± 3.04	43.00 ± 1.94
	Treatment I	54.62 ± 1.33[c]	55.64 ± 4.75[b]	51.78 ± 1.79[b]	44.39 ± 4.57
	Stabilization II	48.20 ± 0.46	43.66 ± 3.66	44.28 ± 1.33	45.67 ± 1.87
	Treatment II	57.34 ± 0.78[b]	61.11 ± 10[a]	55.72 ± 0.75[c]	50.37 ± 1.73[a]
	**Butyrate**	**Inulin**	**b-glucan**	**XOS**	**α-GOS**
1	Stabilization I	33.20 ± 1.10	39.82 ± 0.73	39.82 ± 0.05	29.63 ± 1.65
	Treatment I	33.68 ± 0.45	49.91 ± 4.87[b]	37.62 ± 0.35[a]	52.26 ± 2.26[c]
	Stabilization II	44.19 ± 4.15	32.95 ± 1.37	35.12 ± 1.00	
	Treatment II	62.49 ± 9.70[a]	64.50 ± 7.17[b]	37.62 ± 1.00[b]	
2	Stabilization I	29.28 ± 1.17	31.10 ± 3.40	30.24 ± 2.69	27.56 ± 0.42
	Treatment I	38.59 ± 0.91[c]	33.58 ± 0.74	29.59 ± 2.48	18.6 ± 1.46[c]
	Stabilization II	27.92 ± 0.95	26.71 ± 2.73	33.31 ± 1.35	28.43 ± 1.10
	Treatment II	38.76 ± 0.27[b]	29.30 ± 1.36	29.52 ± 1.00[a]	30.18 ± 1.36

Mean and standard error reported (*n* = 3).
a Significantly different from Stabilization ($p < 0.05$)
b Significantly different from Stabilization ($p < 0.01$)
c Significantly different from Stabilization ($p < 0.001$)

B

Fermentation 3		
**Total SCFAs**	**α-GOS**	**Inulin**
Stabilization	134.21 ± 1.44	114.10 ± 4.25
Treatment	154.97 ± 5.93[a]	138.98 ± 6.51[b]
**Acetate**	**α-GOS**	**Inulin**
Stabilization	71.98 ± 2.73	60.51 ± 1.60
Treatment	71.67 ± 3.65	66.33 ± 1.75[a]
**Propionate**	**α-GOS**	**Inulin**
Stabilization	22.36 ± 1.74	17.50 ± 3.06
Treatment	28.60 ± 1.75[a]	21.24 ± 1.69
**Butyrate**	**α-GOS**	**Inulin**
Stabilization	27.64 ± 1.52	29.79 ± 1.58
Treatment	44.12 ± 0.79[c]	41.03 ± 2.66[b]

Mean and standard error reported (*n* = 3).
a Significantly different from Stabilization ($p < 0.05$)
b Significantly different from Stabilization ($p < 0.01$)
c Significantly different from Stabilization ($p < 0.001$)

Supplementary Table S6: Mean metabolite concentrations (mM) measured by HPLC in effluent samples of stable stabilization and treatment phases in test reactors. **(A)** Mean metabolite concentrations with standard error of effluent sample in Fermentation 1 (donor 3) and 2 (donor 4) during stable stabilization and fiber treatment phases (applying the stability criterion of 20 % variation on 3 consecutive days of a period). (**B**) Mean metabolite concentrations with standard error of effluent sample in Fermentation 3 (D3) (applying the stability criterion of 20 % variation on 3 consecutive days of a period). Significant differences between mean concentration before and at end of treatment period on 0.05 (a), 0.01 (b) and 0.001 (c) level indicated.

A

		Fermentation 1													
**Phylum**	**Family**	**Stab**	**Inulin_I**	**Stab**	**Inulin_II**	**Stab**	**β glucan_I**	**Stab**	**β glucan_II**	**Stab**	**XOS_I**	**Stab**	**XOS_II**	**Stab**	**α GOS_I**
Firmicutes	*Ruminococcaceae*	22%	28%	28%	20%	19%	26%	21%	10%	24%	22%	19%	22%	19%	9%
Bacteroidetes	*Bacteroidaceae*	20%	19%	20%	20%	18%	14%	22%	18%	22%	21%	27%	22%	22%	23%
Firmicutes	*Eubacteriaceae*	18%	8%	12%	11%	10%	23%	7%	20%	7%	8%	6%	6%	10%	14%
Firmicutes	*Lachnospiraceae*	13%	13%	14%	13%	19%	9%	16%	15%	19%	20%	14%	19%	18%	13%
Firmicutes	*Acidaminococcaceae*	18%	11%	12%	10%	19%	16%	10%	13%	13%	10%	13%	15%	12%	14%
Proteobacteria	*Enterobacteriaceae*	0%	10%	5%	12%	1%	2%	11%	7%	1%	7%	7%	3%	11%	11%
Proteobacteria	*Alphaproteobacteria*	1%	1%	0%	1%	2%	0%	3%	2%	2%	3%	7%	0%	2%	0%
Euryarchaeota	*Methanobacteriaceae*	2%	3%	2%	2%	2%	2%	2%	5%	2%	2%	1%	2%	1%	2%
Proteobacteria	*Sutterellaceae*	4%	1%	3%	2%	3%	3%	2%	0%	4%	3%	1%	2%	1%	0%
Firmicutes	*Clostridiaceae*	0%	2%	0%	1%	4%	0%	1%	4%	1%	0%	1%	0%	0%	5%
Firmicutes	*Veillonellaceae*	1%	1%	2%	1%	1%	1%	1%	2%	2%	1%	0%	2%	2%	1%
Firmicutes	*Alphaproteobacteria*	1%	0%	0%	0%	1%	1%	1%	1%	2%	1%	0%	0%	0%	1%
Firmicutes	*Lactobacillales*	0%	0%	0%	2%	0%	1%	1%	2%	0%	0%	1%	0%	0%	1%
Firmicutes	*Lactobacillaceae*	0%	0%	0%	5%	0%	0%	0%	0%	0%	0%	0%	3%	0%	1%

Supplementary Table S7 A: Abundance on family level in fermentation samples before (stab) and at end of dietary fiber suplementation in fermentation 1 with donor 3 (D3) microbiota. For inulin, b glucan and XOS there are two repetitions performed.

B

		Fermentation 2															
Phylum	Family	Stab	Inulin_I	Stab	Inulin_II	Stab	β glucan_I	Stab	β glucan_II	Stab	XOS_I	Stab	XOS_II	Stab	α GOS_I	Stab	α GOS_II
Bacteroidetes	*Prevotellaceae*	57%	52%	53%	58%	26%	61%	54%	58%	55%	57%	54%	52%	49%	49%	58%	55%
Firmicutes	*Ruminococcaceae*	13%	19%	16%	22%	22%	18%	22%	16%	16%	16%	13%	14%	13%	19%	17%	14%
Bacteroidetes	*Bacteroidaceae*	3%	1%	3%	2%	14%	2%	2%	4%	8%	1%	4%	3%	14%	4%	3%	2%
Firmicutes	*Lachnospiraceae*	7%	7%	4%	4%	9%	4%	6%	4%	6%	5%	6%	11%	6%	12%	5%	10%
Proteobacteria	*Desulfovibrionaceae*	2%	2%	4%	2%	6%	1%	2%	2%	3%	3%	3%	2%	3%	2%	2%	2%
Firmicutes	*Eubacteriaceae*	2%	1%	1%	1%	3%	5%	2%	3%	3%	6%	4%	3%	3%	2%	1%	7%
Firmicutes	*Lactobacillales*	3%	4%	6%	2%	6%	1%	2%	3%	2%	5%	6%	6%	3%	5%	6%	4%
Proteobacteria	*Sutterellaceae*	2%	1%	2%	3%	4%	3%	4%	3%	2%	3%	4%	3%	3%	2%	4%	2%
Firmicutes	*Oscillospiraceae*	1%	0%	3%	1%	3%	1%	2%	3%	2%	1%	2%	2%	2%	1%	2%	1%
Firmicutes	*Clostridiales*	0%	0%	1%	0%	3%	0%	0%	0%	1%	0%	1%	0%	1%	0%	0%	0%
Firmicutes	*Veillonellaceae*	1%	1%	1%	1%	1%	1%	2%	1%	1%	2%	1%	1%	1%	1%	1%	2%
Firmicutes	*Lactobacillaceae*	1%	1%	1%	1%	0%	0%	0%	1%	1%	1%	0%	0%	0%	0%	1%	0%
Proteobacteria	*Enterobacteriaceae*	4%	4%	3%	1%	0%	0%	0%	0%	0%	0%	0%	0%	0%	0%	0%	1%
Proteobacteria	*Moraxellaceae*	1%	6%	0%	0%	0%	0%	0%	0%	0%	0%	0%	0%	0%	0%	0%	0%

Supplementary Table S7 B: Abundance on family level in fermentation samples before (stab) and at end of dietary fiber suplementation in fermentation 2 with donor 4 (D4) microbiota. For all dietary fibers there are two repetitions performed.

A

Phylum	Fermentation 3					Fermentation 1				
	Feces_D3	IR_d8	IR_d14	CR_d8	CR_d14	Feces_D3	IR_d12	IR_d19	CR_d12	CR_d19
Firmicutes	50%	67%	68%	82%	84%	51%	58%	56%	65%	55%
Bacteroidetes	31%	29%	28%	10%	6%	25%	27%	26%	23%	28%
Proteobacteria	5%	1%	2%	7%	7%	3%	14%	18%	11%	15%
Euryarchaeota	6%	2%	1%	1%	1%	9%	1%	1%	0%	1%
Actinobacteria	4%	2%	1%	1%	1%	6%	0%	0%	0%	0%
Verrucomicrobia	4%	0%	0%	0%	0%	6%	0%	0%	0%	0%

B

		Fermentation 3					Fermentation 1				
Phylum	Family	Feces_D3	IR_d8	IR_d14	CR_d8	CR_d14	Feces_D3	IR_d12	IR_d19	CR_d12	CR_d19
Firmicutes	*Ruminococcaceae*	16.4%	16.0%	22.5%	38.7%	43.2%	20.9%	18.8%	19.0%	20.8%	6.6%
Firmicutes	*Acidaminococcaceae*	0.6%	22.9%	15.7%	11.8%	18.8%	0.3%	12.6%	10.3%	15.4%	11.9%
Firmicutes	*Eubacteriaceae*	3.4%	14.1%	17.0%	19.1%	9.6%	4.3%	8.5%	7.7%	4.9%	16.2%
Proteobacteria	*Enterobacteriaceae*	0.3%	0.0%	0.0%	6.1%	7.1%	0.1%	9.7%	15.0%	5.3%	8.9%
Firmicutes	*Lachnospiraceae*	11.0%	8.7%	8.6%	8.7%	6.5%	7.3%	13.2%	13.0%	13.9%	14.6%
Bacteroidetes	*Bacteroidaceae*	21.7%	29.0%	28.3%	9.9%	6.4%	16.9%	26.9%	25.6%	23.3%	28.3%
Euryarchaeota	*Methanobacteriaceae*	6.0%	1.9%	0.6%	0.8%	1.3%	8.7%	0.7%	0.6%	0.1%	1.4%
Firmicutes	*Oscillospiraceae*	8.9%	1.0%	0.6%	0.6%	0.5%	6.8%	0.9%	0.7%	0.2%	0.6%
Verrucomicrobia	*Verrucomicrobiaceae*	4.2%	0.0%	0.0%	0.0%	0.0%	6.1%	0.0%	0.0%	0.0%	0.0%
Bacteroidetes	*Rikenellaceae*	4.4%	0.0%	0.0%	0.0%	0.0%	6.1%	0.0%	0.1%	0.0%	0.1%

Supplementary Table S8: Summary microbial phyla obtained by V4 region 16S amplicon sequencing within non-treated PolyFermS reactors and their respective fecal inoculum of donor 3. **B**:Summary of most abundant (>5%) microbial families obtained by 16S V4 region amplicon sequencing within non-treated PolyFermS reactors with donor 3 (D3) microbiota. Inoculum reactor (IR) and control reactor (CR) of fermentation 1 on day 12 and day 19 and of fermentation 3 on day 8 and day 14.

		Fermentation 3				Fermentation 1					
**Phylum**	**Family**	**Stab**	**Inulin_I**	**Stab**	**a GOS_I**	**Stab**	**Inulin_I**	**Stab**	**Inulin_II**	**Stab**	**a GOS_I**
Firmicutes	*Ruminococcaceae*	41%	48%	24%	31%	22%	29%	28%	19%	19%	9%
Bacteroidetes	*Bacteroidaceae*	6%	6%	22%	26%	20%	19%	21%	21%	21%	23%
Firmicutes	*Acidaminococcaceae*	16%	15%	17%	16%	17%	11%	12%	10%	13%	13%
Firmicutes	*Lachnospiraceae*	14%	12%	9%	10%	14%	14%	14%	13%	18%	13%
Firmicutes	*Eubacteriaceae*	15%	9%	17%	10%	18%	8%	12%	11%	10%	14%
Firmicutes	*Veillonellaceae*	2%	2%	2%	1%	1%	2%	2%	1%	2%	1%
Firmicutes	*Oscillospiraceae*	1%	1%	1%	1%	0%	0%	1%	0%	0%	0%
Firmicutes	*Lactobacillaceae*	1%	2%	1%	1%	0%	0%	0%	4%	0%	1%
Euryarchaeota	*Methanobacteriaceae*	1%	1%	2%	1%	2%	3%	2%	2%	1%	2%
Actinobacteria	*Bifidobacteriaceae*	1%	1%	0%	0%	0%	0%	0%	0%	0%	3%
Firmicutes	*Clostridiaceae*	1%	1%	1%	0%	0%	2%	0%	1%	0%	5%
Firmicutes	*Other*	0%	2%	0%	0%	0%	0%	0%	2%	0%	1%
Proteobacteria	*unclassified*	0%	0%	0%	0%	1%	1%	0%	1%	2%	0%
Proteobacteria	*Sutterellaceae*	0%	0%	1%	0%	4%	1%	3%	2%	1%	0%
Proteobacteria	*Enterobacteriaceae*	0%	0%	0%	0%	0%	10%	4%	12%	11%	11%

Supplementary Table S9 Abundance on family level in fermentation samples before (stab) and at end of dietary fiber suplementation in fermentation 1 and 3 (donor 3).

Supplementary figures

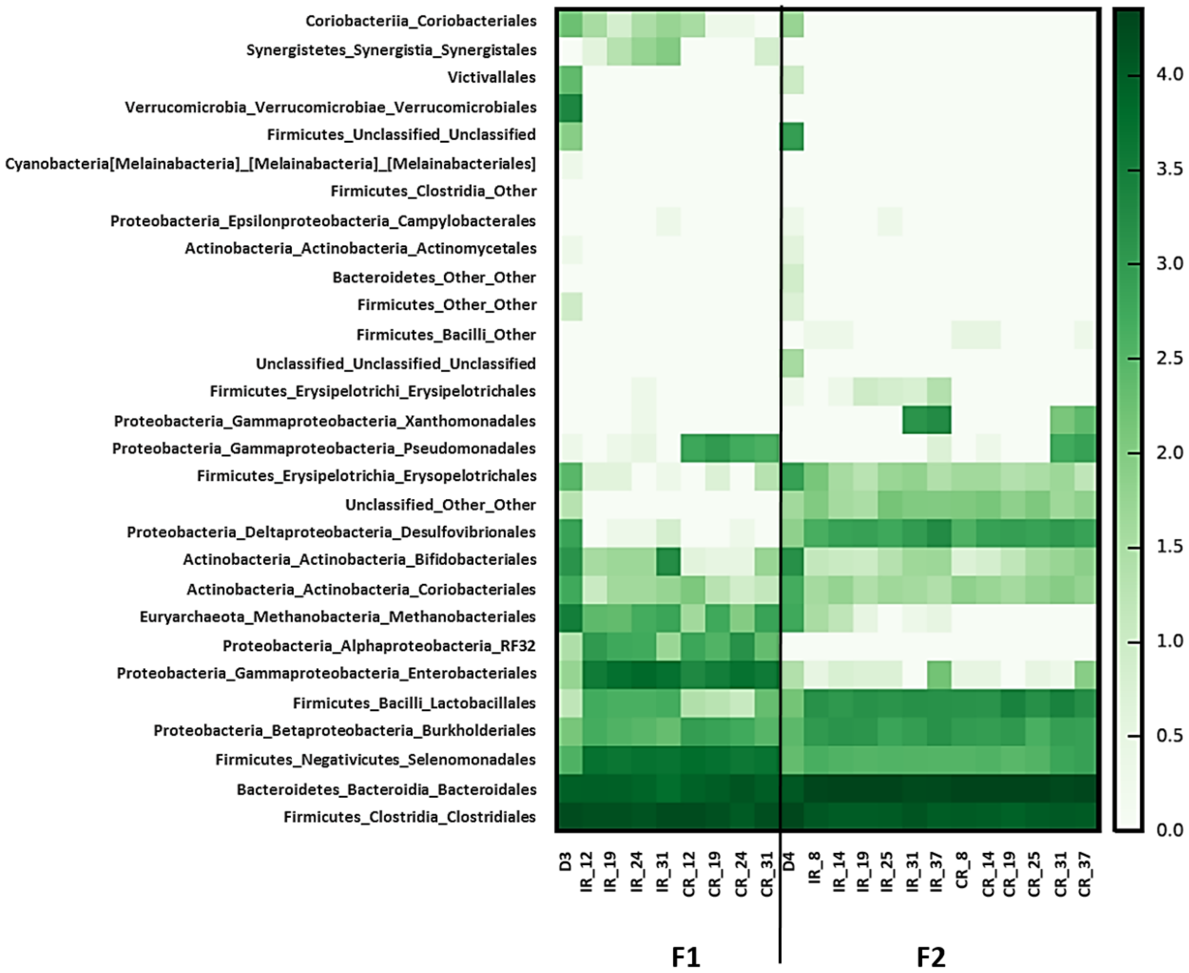

Supplementary Figure S1: Heatmap depicting the most abundant OTUs (> 1 %) at order level in the fecal (donor 3 and donor 4) and PolyFermS microbiota of inoculum reactor (IR) and control reactor (CR) along both fermentations. Heatmap was constructed using normalized abundance of each OTU.

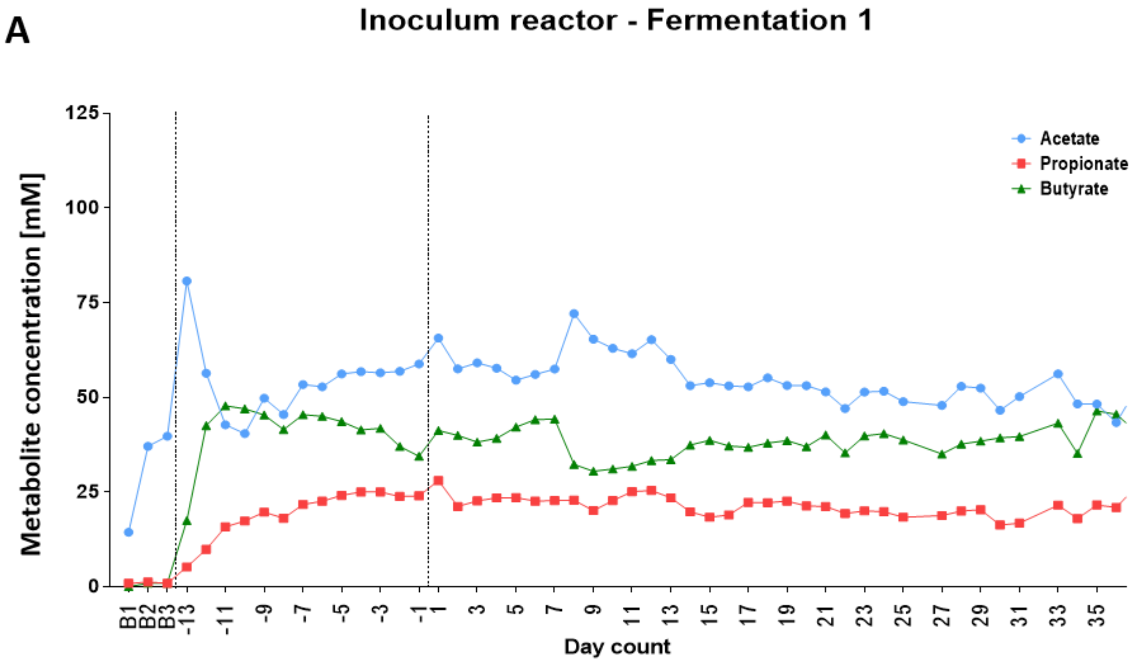

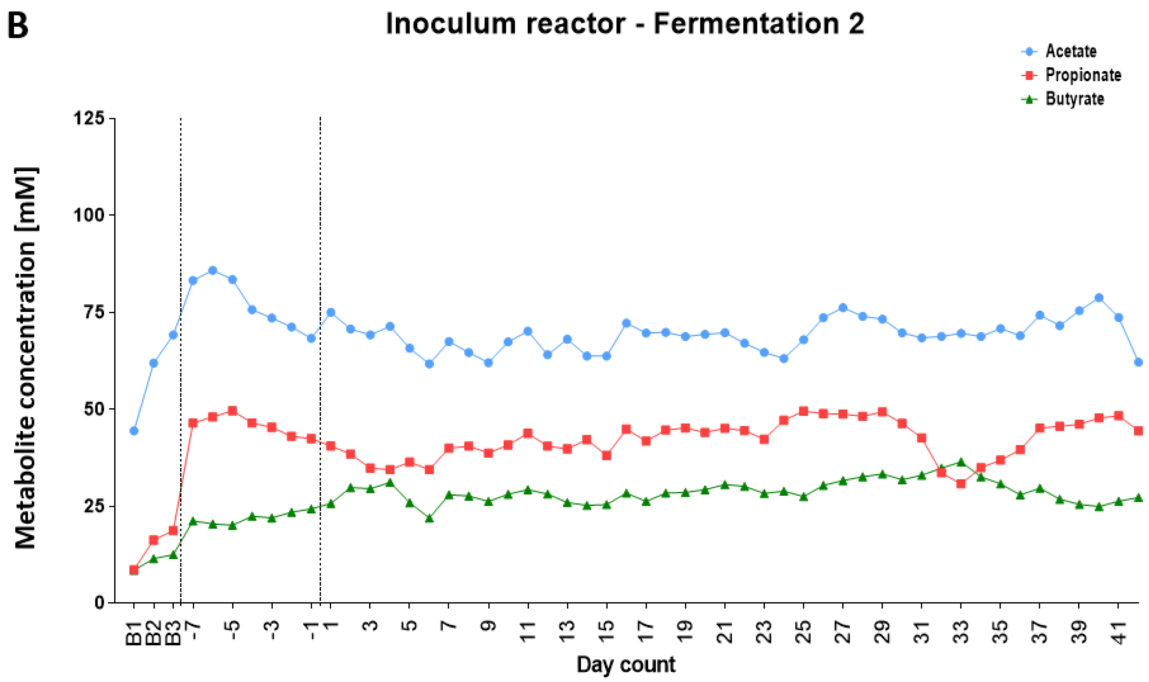

Supplementary Figure S2: Daily SCFA concentrations in fermentation effluents of inoculum reactor (IR) (**A & C**) of both PolyFermS fermentations measured by HPLC. Colonization: three consecutive batch fermentations for bead colonization. IR stabilization: stabilization period in continuous mode to reach steady-state

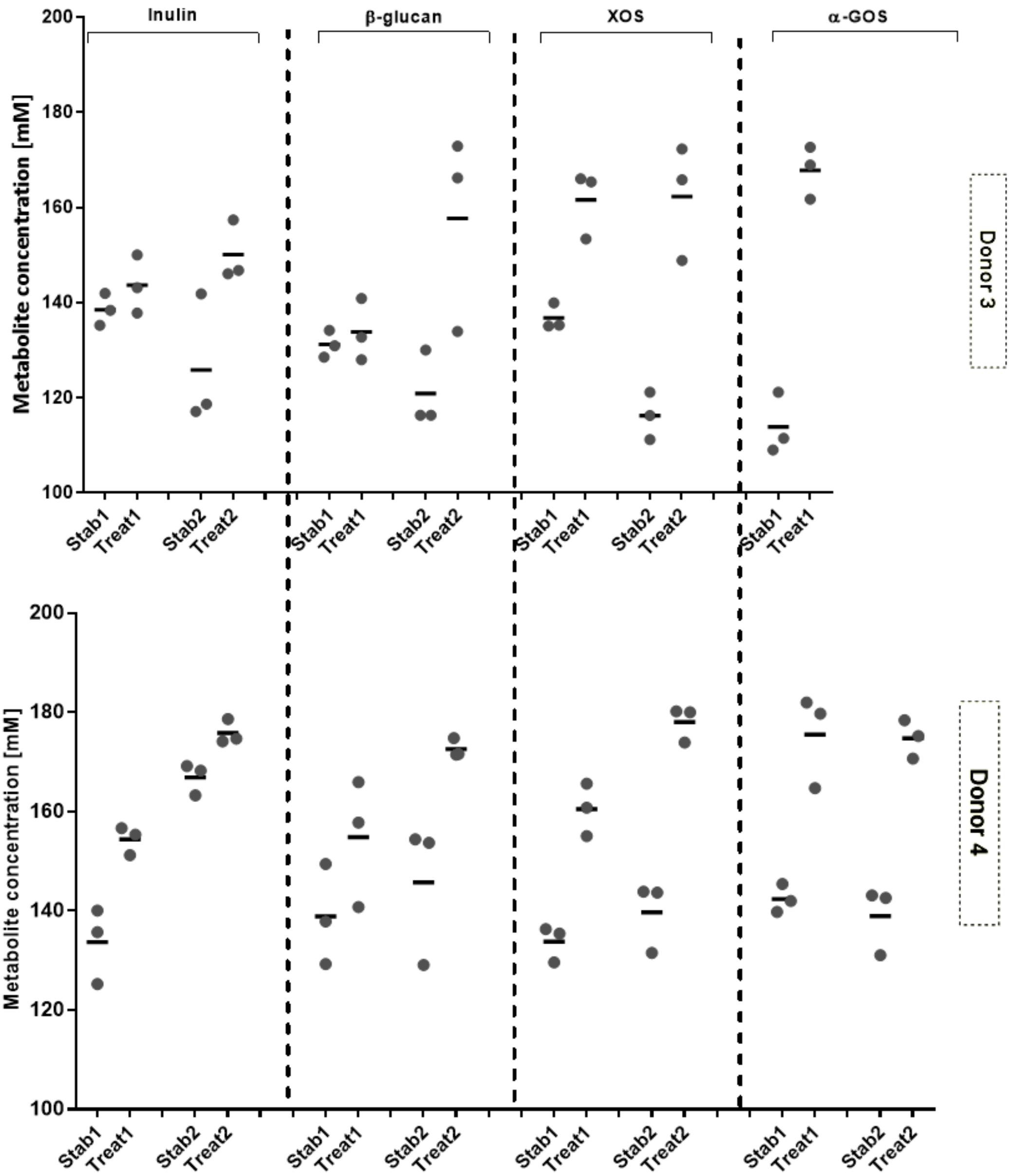

Supplementary Figure S3: Treatments with dietary fibers result in higher Total SCFA productions. Mean (black horizontal line) from three consecutive measurements of total SCFAs (mM) in the respective reactor at end of stabilization and treatment phase for fermentation 1 (Donor 3) and fermentation 2 (Donor 4). Two replicates are shown for each dietary fiber treatment, except α-GOS in fermentation

1.

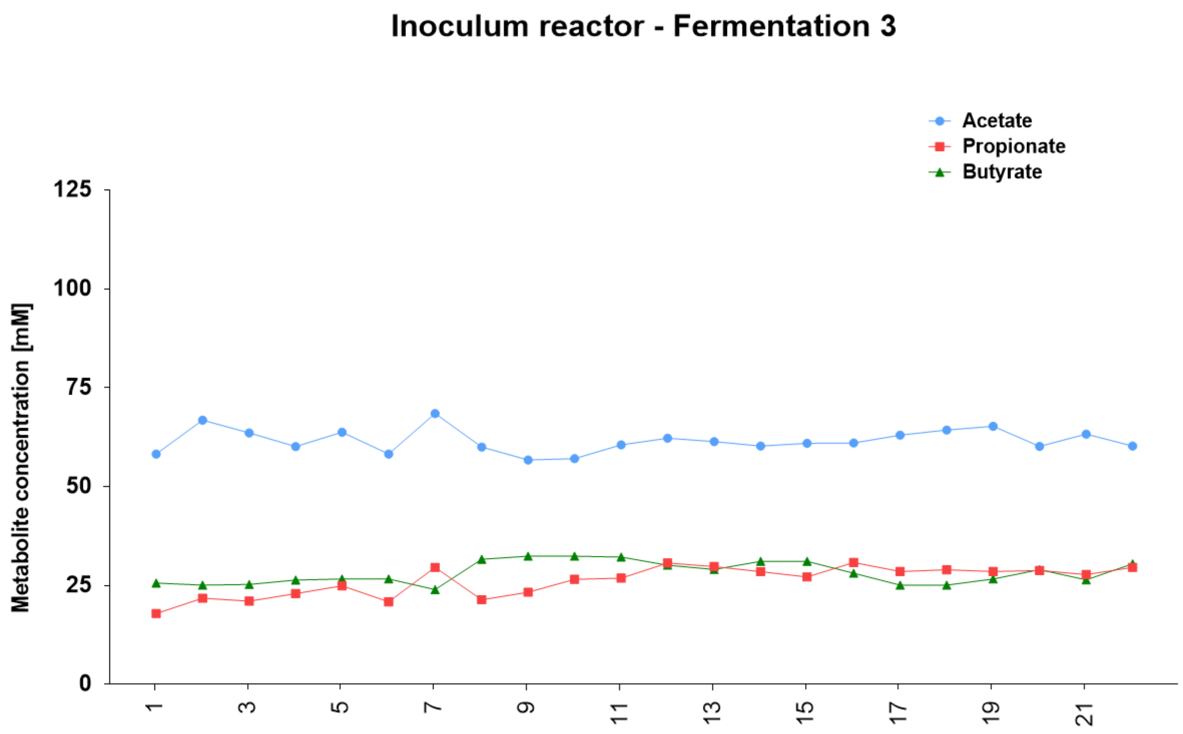

Supplementary Figure S4: Daily SCFA concentrations in fermentation effluents of inoculum reactor (IR) in Fermentation 3 (donor 3) measured by HPLC.

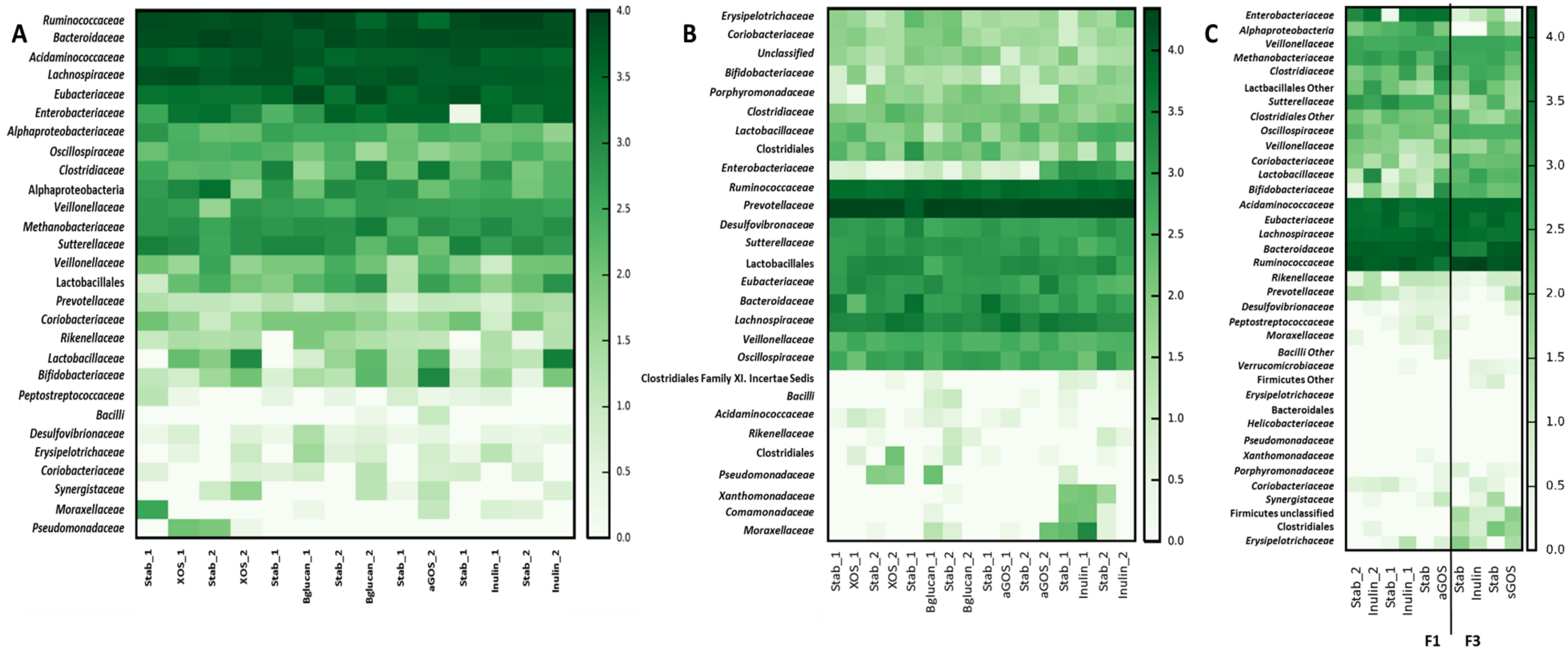

Supplementary Figure S5: Heatmap depicting abundance of the most abundant OTUs (> 1 %) on family level in fermentation microbiota at end of stabilization and treatment periods. Heatmap was constructed using normalized abundance of each OTU. **A**: F1 (D3), **B**: F2 (D4), **C**: F1+F3 (D3); F, fermentation; D, donor.
